# Automatic Selection of Search Parameter Values for Mass Spectrometry-Based Search Engines

**DOI:** 10.1021/acs.jproteome.5c00641

**Published:** 2026-01-24

**Authors:** Yehia M. Farag, Henrik Ø. Søgaard, Harald Barsnes

**Affiliations:** † Proteomics Unit, Department of Biomedicine, 1658 University of Bergen, Bergen 5020, Norway; ‡ Computational Biology Unit, Department of Informatics, University of Bergen, Bergen 5008, Norway

**Keywords:** proteomics, mass spectrometry, data processing, protein identification, search engines, autoselection, search parameters

## Abstract

A crucial step in processing mass spectrometry-based proteomics data is identifying and quantifying the proteins in the sample. While the existing search engines can easily match tandem mass spectra to peptide sequences, selecting the most appropriate search parameters can often be challenging and time-consuming due to the diversity of the data sets and the long list of available parameter values to choose from. This study introduces QuickSearchProt—an algorithm aimed at assisting in the selection of search parameter values across search engines, considering not only the data set specifications but also the properties of the search algorithms. By relying on a small representative subset of the spectra, the algorithm can process most data sets within minutes, largely independent of the size of the original data set. The current implementation supports two common search engines, X! Tandem and Sage, and is designed to process data-dependent acquisition (DDA) proteomics data sets but due to its adaptability and scalability can easily be extended to additional search engines. QuickSearchProt, including a graphical user interface, the complete source code, and additional details are freely available at http://www.github.com/barsnes-group/QuickSearchProt.

## Graphical abstract

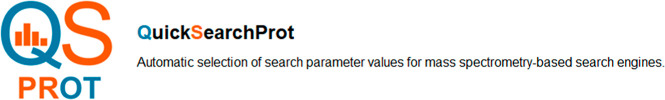

## Introduction

Mass spectrometry-based proteomics is a high-throughput technology widely used for identifying and quantifying thousands of proteins in biological samples.^1^ It determines the mass-to-charge (*m*/*z*) ratios and signal intensities of protein fragments, generating experimental mass spectra. The key to identifying the resulting spectra is so-called proteomics search engines, which interpret mass spectra by mapping them to peptide sequences.^2^

Numerous search engines have been developed to match tandem mass spectra to peptide sequences, e.g., X! Tandem,^3^ Sage,^4^ and MS-GF+.^5^ However, most search engines require a long list of search parameters, including common parameters such as enzyme type and specificity, mass tolerances, and post-translational modifications (PTMs), in addition to more advanced search parameters specific to each search engine, e.g., the minimum ion index for Sage. Choosing the correct values for all these parameters is crucial for protein identification. For example, using inappropriate values can negatively impact the search results, while using the most suitable values may both enhance the number of detected spectra and increase the confidence level of the mapped peptides.

Selecting the most suitable parameter values can, however, be challenging, especially if the data set specifications are unknown or the user is inexperienced. Fortunately, attempts have been made to address this challenge, offering fully or partially automated parameter selection. For example, tools like Preview^6^ assist with selecting various parameter values, including precursor and fragment mass tolerances, digestion specificity, and PTMs, in addition to the recalibration of mass over- charge measurements. Similarly, Param-Medic^7^ focuses on estimating precursor and fragment mass tolerances, while PTMselect^8^ aims to identify the best combinations of proteases for comprehensive coverage of PTMs for both targeted and untargeted protein analysis in a given data set.

An important limitation of the currently available tools is that they typically focus on specific parameters, individually or in combination, and none of them offer a customizable algorithm to effectively address the remaining search parameters. Furthermore, they do not consider the underlying details of the search algorithms or provide suggestions for advanced parameters unique to each search engine.

In this study, we investigate and evaluate the performance of a more adaptable algorithm called QuickSearchProt designed to assist in discovering appropriate search parameter values across various search engines. The algorithm takes into account both the data set specifications and the search algorithms used. QuickSearchProt currently supports all search parameters for X! Tandem and Sage, but they can easily be extended to additional search engines.

## Methods

The five key steps of the algorithm are as follows: (i) reduce the spectrum input to a smaller subset, (ii) reduce the size of the sequence database, (iii) perform searches using a targeted parameter, (iv) compare the results and choose the best value for the targeted parameter, and (v) continue with additional parameters, if any. Figure 1 provides an overview of the workflow.

**Figure 1:**
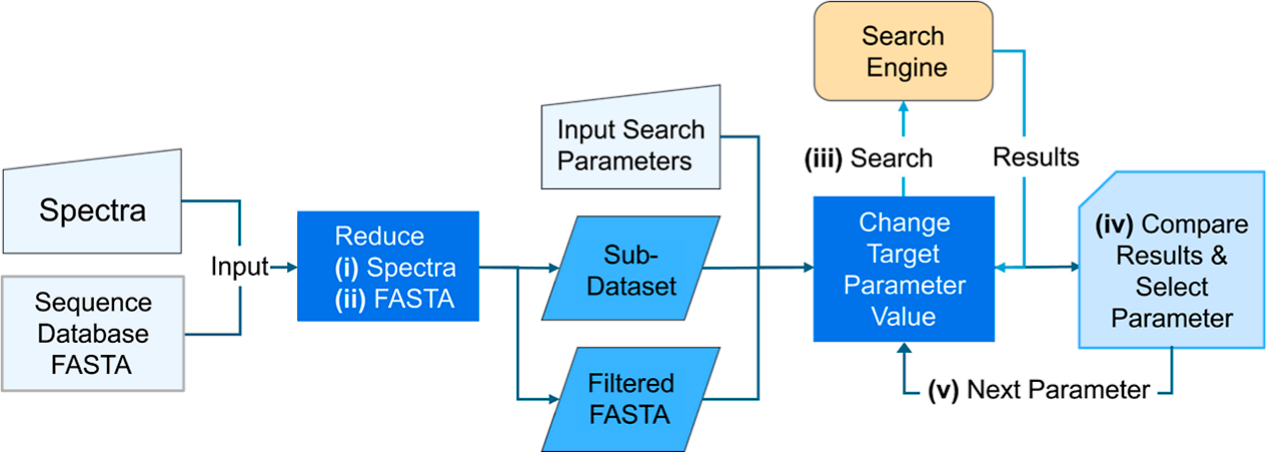
Overview of the QuickSearchProt workflow.

### Spectrum Subset Selection

Subset size selection: To ensure efficient processing and reliable FDR estimation, the subset size was determined based on the data set size and the search engine used. For X! Tandem: if the input file contains fewer than 1500 spectra, the subset size is set to ≤1500, provided that the total number of spectra is below this threshold. For data sets exceeding 1,00,000 spectra, the subset size is increased to 2000. For Sage: due to the searching speed, the minimum size of the subset was expanded to 3000 (if the data set contains fewer than 3000 spectra, the subset size is capped at the data set size). The suggested subset size was determined by testing different subset sizes with the two search engines (500, 1000,1500, 2000, 3000, 5000, 10,000, and 20,000 spectra; results not shown) and demonstrating that they provide enough data to perform FDR evaluation and enough identifications for selecting appropriate parameter values while also maintaining an acceptable processing speed.

Sectioning strategy for full data set coverage: To ensure comprehensive coverage of the input file, the data set was divided into multiple sections based on the total number of spectra: <2000 spectra: 4 sections. 2000–10,000 spectra: 4 to 20 sections, each containing 500 spectra. > 10,000 spectra: 20 sections, with each section containing approximately (total spectra/20 spectra).

Confidence-based filtering using DirecTag:^9^ To refine the data set and retain high-confidence identifications, a two-step filtering process was applied using DirecTag: Spectra with low-confidence tags (*e*-value ≥ 0.01) were excluded. Within each section, the distribution of high-confidence spectra (*e*-value < 0.01) was preserved to maintain representative coverage.

Subset reduction with sectionwise ratio preservation: After filtering, a reduction procedure was applied to select spectra according to the targeted subset size: For each section, the number of high-confidence spectra was assessed. A section-specific sampling interval (“every-*n*”) was computed to maintain the original ratio of confident spectra in each section. Spectra were uniformly sampled using this interval, ensuring consistent sectionwise coverage while achieving the desired subset size.

### Sequence Database Filtering

The filtering step for the sequence database starts by retaining only sequences with experimental evidence at the protein level, i.e., protein existence code PE = 1 in UniProt.^10^ The spectrum subset is then searched with Novor,^11^ generating a set of potentially corresponding peptide sequences. Protein sequences containing the subset-generated peptide sequences are then retained, creating a smaller protein sequence database that includes only the matched protein sequences. Figure 2 summarizes the subset generation and database reduction steps.

**Figure 2:**
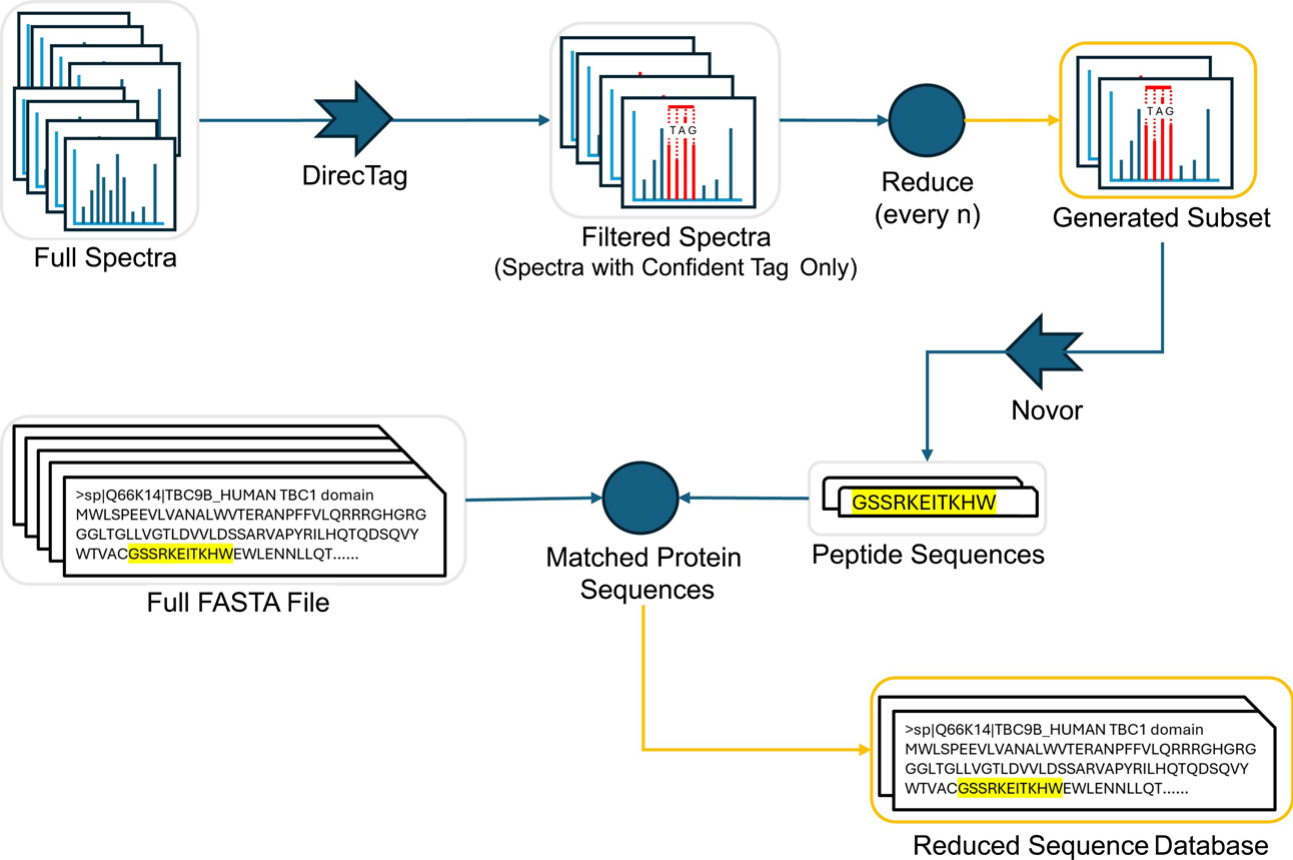
Key steps for reducing a data set and sequence database into a refined subset and filtered sequence database in QuickSearchProt.

### Parameter Selection

The automatic value-selection for a targeted parameter is carried out in two steps. First, an initial search, termed the reference search, is conducted using the default value for the targeted parameter. Multiple additional searches are then performed using the other supported values: categorical values in the case of discrete parameters and prespecified steps for numerical ones. Each search is conducted with a different supported value for the targeted parameter and compared to the reference search result. For example, when targeting the digestion enzyme parameter, the default value of “trypsin” is used for the reference search, and additional searches using the other enzyme options are then conducted and compared against the reference result.

A target-decoy strategy^12^ is applied to control the false positives in the generated results. This involves concatenating decoy sequences (reversed sequences) to the sequence database (after filtering). The identified decoy PSM scores are then used to estimate the false discovery rate (FDR). A threshold is calculated for the search results to achieve a 1% false discovery rate (FDR) to ensure high accuracy and minimize false positives. Only the data above this threshold are retained, while the rest is disregarded. This step is essential, especially for parameters potentially leading to increased false positive identifications, e.g., fragment and precursor tolerance, and PTMs.

### CScore

Results from the individual searches are compared using the comparison score (CScore), providing a quick evaluation of the hyperscores for the peptide-to-spectrum matches (PSMs). PSM hyperscore is a matching score that evaluates the similarity of an experimental spectrum to a theoretical one from a specific peptide.^3^ This score is calculated based on several factors, including correlation factors, the matched ions number to a peptide sequence, k-similarity statistics, and other probability factors. Initially, it is affected by the intensity of sequence-specific ions present in the mass list, along with the database algorithm parameters.^3^ By using hyperscores generated from various search engines, QuickSearchProt incorporates the unique characteristics of each algorithm into its scoring process. The distinct properties of each engine directly influence the generated hyperscore, which, in turn, shapes the final CScore—allowing the system to reflect the specific behavior of each search engine in its overall evaluation.

The following equation is used to calculate the CScore:

CScore=shareddatascore+gaineddatascore−lostdatascore

CScore consists of three parts: (i) shared data score, an evaluation of the shared spectra between the reference and test searches; (ii) gained data score, an evaluation of the uniquely identified spectra for the test search; and (iii) lost data score, an evaluation of the uniquely identified spectra for the reference search.

First, the reference data hyperscores are sorted, and the first quartile, the median, and the third quartile are computed, thus dividing the reference data into four sections. The sections are then ranked from 1 to 4, reflecting the gradual improvement in result quality as a higher hyperscore indicates greater similarity between the observed and theoretical spectra.^3^ Each PSM hyperscore from the test data is then mapped to the corresponding quartile range from the reference, assigning it a rank. In this way, all PSM hyperscores from both the reference and test data are converted to rank values.

The shared data score measures the change in paired PSMs, i.e., PSMs for the same spectrum from the reference and test data that share the same search parameter values, except for the targeted parameter. It is calculated by subtracting the rank of the test PSM from the rank of the reference PSM, summed across each pair of spectra. To minimize the impact of minor fluctuations, data that shift between adjacent quartile ranges are disregarded.

$$shared\;data\;score=\sum \left({PSM\;rank}_{paired-test}-{PSM\;rank}_{paired-reference}\right)$$

The gained data score quantifies the impact of newly identified PSMs in the test search:

$$gained\;data\;score=\sum {PSM\;rank}_{unique-test}$$

The lost data score quantifies the impact of the PSMs no longer identified in the test search:

$$lost\;data\;score=\sum {PSM\;rank}_{unique-reference}$$

### Order of the Search Parameters

Users can specify their preferred order for the targeted parameters; however, a default order for each search engine is provided, as detailed in the configurations.json file found at the algorithm’s GitHub page, including a full list of X! Tandem and Sage parameter orders. The default order of the search parameters in QuickSearchProt is fixed and was decided based on three key factors: (i) the natural order of the parameters, (ii) their impact on the search results, and (iii) their influence on the search speed. For example, digestion type, enzyme, enzyme specificity, and the maximum number of missed cleavages naturally follow each other. Furthermore, parameters that have a large effect on the output were placed earlier to reduce any compound errors that can affect the results. For example, digestion enzymes have the greatest impact on the result; hence, selecting the most suitable digesting enzyme comes early in the order.

The effect of these parameters on the final results was measured using the CScore range; i.e., the wider the range, the earlier the parameter appears in the order. The effect on the processing speed also was also considered. For example, introducing PTMs early will greatly impact the speed of all subsequent searches. However, some parameters have to come after the PTMs since PTMs directly impact their values, e.g., the Max Variable Modification and Peptide Mass parameters in Sage or the Potential Modifications and Point Mutation parameters in X! Tandem.

### Test Data Sets and Performance Evaluation

The algorithm was tested on six data sets containing a number of spectra ranging from 8000 to 1,50,000. The spectrum files and the user-defined search parameters were collected from the selected projects available in PRIDE.^13^ The accession numbers for the selected projects are PXD000674 (data set 1), PXD000561 (data set 2), PXD001468 (data set 3), PXD047036 (data set 4), PXD009340 (data set 5), and PXD001250 (data set 6). In addition, suitable protein sequence databases were downloaded from UniProt.^10^ The six proteomics data sets selected for benchmarking exhibit strong methodological consistency while offering diverse biological contexts and analytical depth. All data sets employ data-dependent acquisition (DDA), as the algorithm relies on SearchGUI^14^ to conduct the searches, which currently only supports DDA-based data sets. These data sets were generated using high-resolution Orbitrap-based mass spectrometers, ensuring consistent spectral quality across experiments. Trypsin is the primary proteolytic enzyme used throughout, reflecting the standard practice in shotgun proteomics. All tested data sets include carbamidomethylation of cysteine as a fixed modification and oxidation of methionine as a variable one. Despite these shared foundations, the data sets vary significantly in size and post-translational modification (PTM) complexity—ranging from specific cell-line studies such as PXD009340 (Jurkat T-cells) to broader tissue-wide proteome maps involving multiple tissues and regions, e.g., PXD000561 and PXD001250.

Among the six benchmarking data sets, PXD001468 stands out as a highly PTM-enriched resource, purpose-built to explore the complexity of post-translational modifications in shotgun proteomics, making it particularly relevant for benchmarking modification search strategies. The search and analysis of the data sets were conducted using SearchGUI and PeptideShaker.^15^

### Search Mode

Full Data mode: Refers to the automatically selected search parameters obtained by conducting searches using the complete input files—including all spectra and the full sequence database—providing a comprehensive baseline for comparison.

Subset mode: Refers to the automatically selected search parameters derived from using a reduced data set and a filtered sequence database as input. This mode aims to balance speed and accuracy by minimizing data volume while retaining representative information.

PRIDE mode: Applies user-defined search parameters as listed on the corresponding data set page in PRIDE, serving as an external reference for benchmarking and validation.

Development and comprehensive testing were performed on a personal computer, specifically a Dell Precision 5550, equipped with an Intel­(R) Core­(TM) i9-10885H CPU running at 2.40 GHz with 8 cores and 16 logical processors and 32 GB of RAM. The system currently requires Java 19 and Visual C++ Redistributable for Visual Studio 2012 Update 4. A minimum of 1 GB of available memory is recommended to ensure stable performance; however, allocating more memory when working with larger data sets is advised to maintain efficiency and avoid potential slowdowns. QuickSearchProt supports the MGF file format for spectral data input and the FASTA format for the protein sequence database. Additionally, it accepts search parameter files in the .par format, which can be exported directly from SearchGUI. The complete source code and additional details are freely available at http://www.github.com/barsnes-group/QuickSearchProt.

## Results and Discussion

The work resulted in QuickSearchProt, an automated algorithm for selecting search parameter values across proteomics search engines. It features a user-friendly graphical interface to manage inputs/outputs, configure parameters and engines, and define subset sizes, streamlining workflows and improving reproducibility (Figure 3).

**Figure 3:**
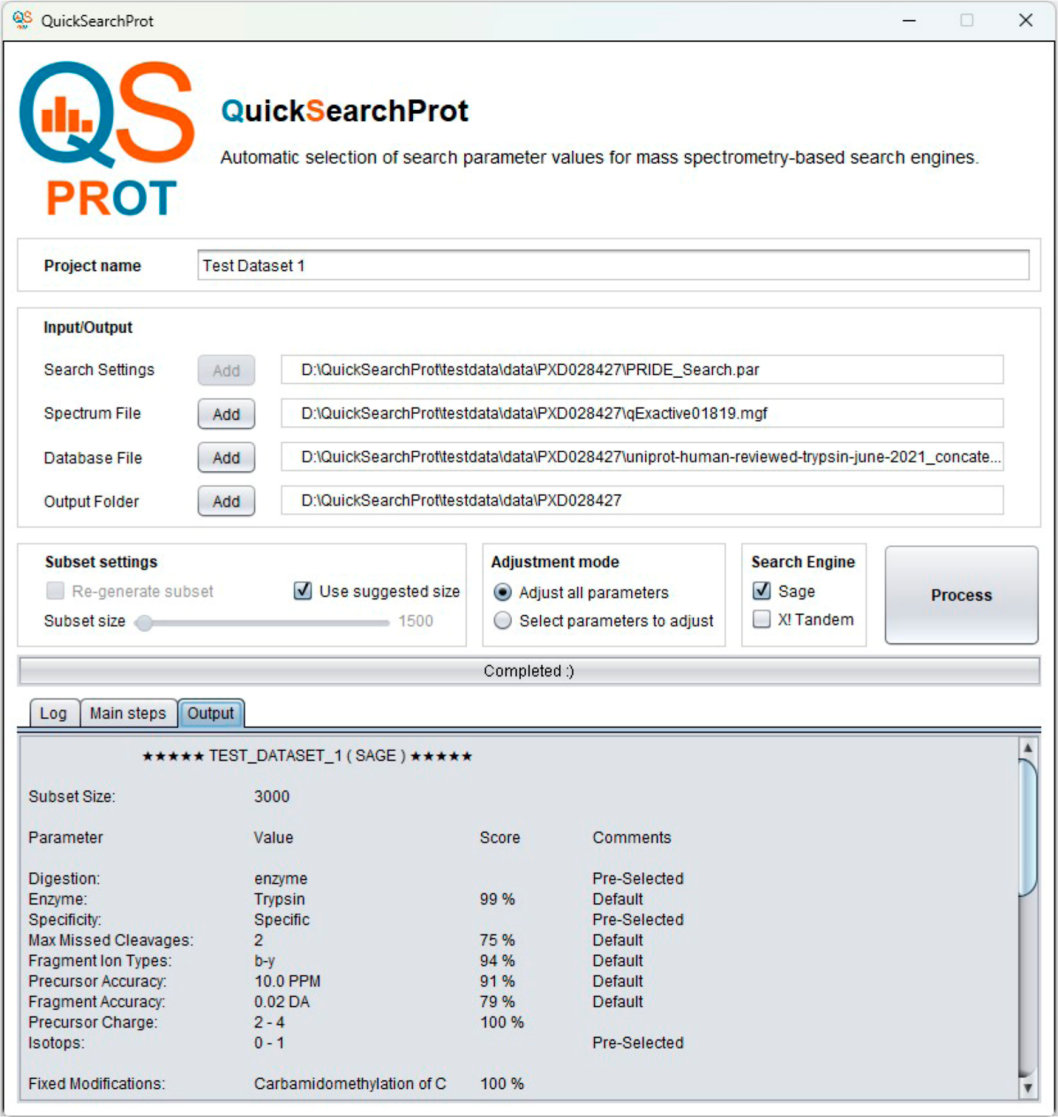
Screenshot of QuickSearchProt, showing input controls, search parameter settings, and the three output panels.

### Data Reduction Strategy and Its Effects

Randomly reducing the number of spectra could significantly affect the data representation, which would make it more difficult to arrive at suitable search parameter values. This can be addressed by targeting only potentially identifiable spectra, thus reducing the number of spectra while minimizing the impact on the data representation. The analysis of the test data sets shows that on average, 97% of the selected spectra with confident tags were successfully identified under appropriate search conditions (data not shown), thus providing a quick and easy way to arrive at a smaller but still representative data set. When dealing with larger data sets, an additional filtering step was, however, required to further speed up the process. By selecting only a subset of the filtered spectra, specifically every *n*th spectrum from different sections of the data, the desired speed could be achieved while preserving data integrity.

The size of the protein sequence database was also a factor. While large sequence databases will greatly increase the processing time, making it too small can, on the other hand, severely impact the identification rate. Reducing the size of the database while still retaining potential protein-matching sequences was therefore essential to (i) minimize potentially harmful filtering effects, (ii) maintain an acceptable identification rate, and (iii) reduce the processing time. By matching potential peptide sequences generated by Novor from the spectra included in the subset with sequences in the original database, the size of the sequence database could be reduced while maintaining an acceptable identification rate necessary for the automatic selection of search parameter values.

A comparison of the performance obtained using the reduced data (Subset mode), with those obtained from the original data sets and sequence database (Full Data mode), revealed substantial improvements in processing speed (Supporting Information Tables S1 and S2). For example, with a medium-sized data set, e.g., data set 4 (PXD047036) with around 50,000 spectra, the time required to select all of the parameter values in Subset mode with Sage was four minutes, compared to 44 min in Full Data mode. This difference becomes even more pronounced with X! Tandem, which required four minutes in Subset mode, compared to 3.5 h in Full Data mode.

Overall, the time required for selecting parameter values in Subset mode was consistently faster, even with small data sets and a fast search engine like Sage. Furthermore, there was a high level of agreement in the individual parameter values selected across both modes. Most parameter values were consistent; however a few differed, including variable PTMs and certain filtering parameters, including spectrum dynamic range, fragment mz, and the maximum and total number of peaks filter in X! Tandem and Sage. These parameters were more difficult to optimize in Subset mode compared to the Full Data mode.

Figure 4 illustrates the intersection of identified PSMs with the same peptide sequence from both modes (middle segment), highlighting PSMs that are gained (lower segment) or lost (top segment) when using Subset mode instead of Full Data mode. The intersections of identified PSMs cover a large part of the data in all data sets, indicating a high level of agreement regarding most search parameters. This shows that the performance in Subset mode is efficient regarding processing time while still achieving very similar results.

**Figure 4:**
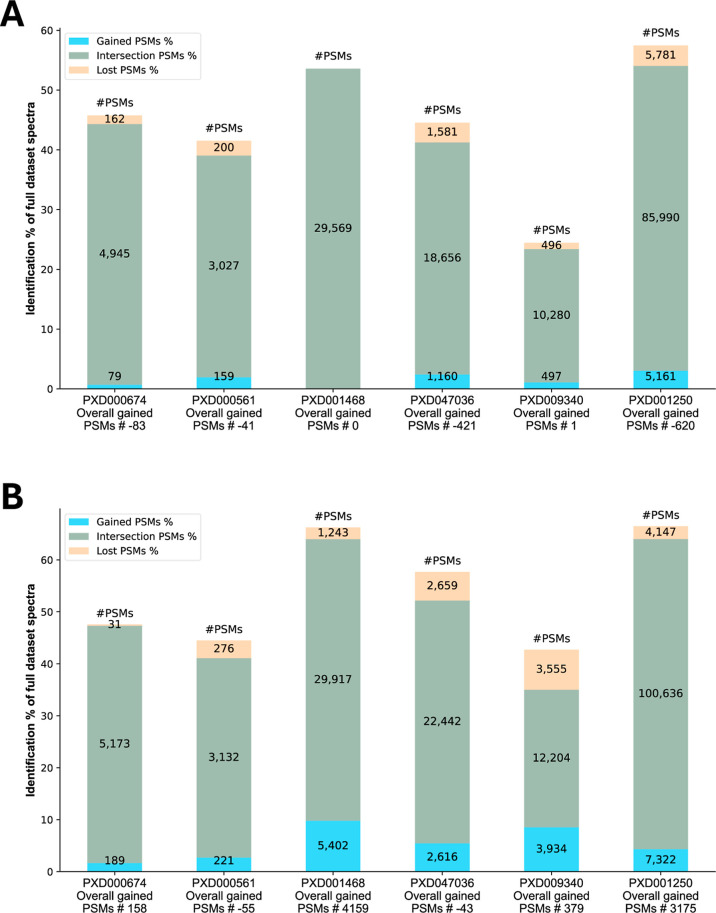
(A) Comparison of Subset mode vs Full Data mode for X! Tandem. (B) Comparison of Subset mode vs Full Data mode for Sage. The *y*-axis indicates the percentage of spectra identified from the complete spectrum file.

There will always be a trade-off between speed and accuracy. Using larger subsets and sequence databases will increase processing time but also offer better coverage of spectral and sequence properties, improving false discovery rate (FDR) estimation and overall result accuracy. Conversely, using smaller subsets and reduced sequence databases accelerates processing but may risk omitting relevant information, potentially compromising the accuracy of the output.

The size of the data sets may also impact specific parameters, particularly those related to PTMs, where more data generally indicate a greater likelihood of identifying a wider range of PTMs. Similarly, filtering parameters, such as minimum and maximum number of peaks per spectrum in Sage, are also affected, with Full Data mode tending to select a broader range compared to Subset mode, but overall, QuickSearchProt consistently maintained acceptable performance across key metrics. Notably, small fluctuations in subset size—within ±10% of the target—yielded robust and stable results (data not shown). This resilience is attributed to the sectionwise coverage strategy, which ensures proportional representation and mitigates the impact of minor subset size variations on the final output.

While including more spectra can be beneficial for certain types of parameters, it often also comes at the cost of lowering the overall data quality. The full data set usually contains spectra that vary in quality, where the lower-quality spectra introduce noise and result in false positives, thus affecting the parameter selection accuracy. By reducing the input data, we aim to select only potentially identifiable data, thus improving the overall quality of the spectra in the subset. A higher-quality subset of data can therefore perform similarly to the full data set or even outperform it. For example, for data set 6 (PXD001250) using Sage, most of the selected parameters were identical in Subset mode and Full Data mode, except for one filtering parameter: fragment mz range. Here, the advantages of using a high-quality spectrum subset become clear, as it selects more suitable parameters, fragment mz range between 175 and 1750 in the Subset mode, compared to 150 and 2,000 in Full Data mode, resulting in improved outcomes for the Subset mode.

### Single-Parameter Optimization with CScore

To assess the single parameter selection performance, a series of evaluations were conducted by (i) selecting test parameters and performing multiple searches using the full data set while keeping all other conditions fixed and varying only the selected parameter per search, (ii) recording the scores for each parameter value, and (iii) comparing the scores with the number of confident PSMs obtained from equivalent full-data searches.

Figure 5 illustrates the CScore performance for a single parameter—digestion enzyme—on data set 1 (PXD000674) using Sage. The results show a near-perfect alignment between the CScores and the number of confident PSMs for each enzyme (Pearson correlation coefficient: 0.9994, *p*-value < 0.00001), confirming the suitability of the selected value. Notably, the complete process took 30 s, compared to 15 min required to run 16 separate enzyme-specific searches, even for a relatively small data set. Full scoring details and processing times are provided in Supporting Information Table 3, with a comparative summary available in Supporting Information Table 6.

**Figure 5:**
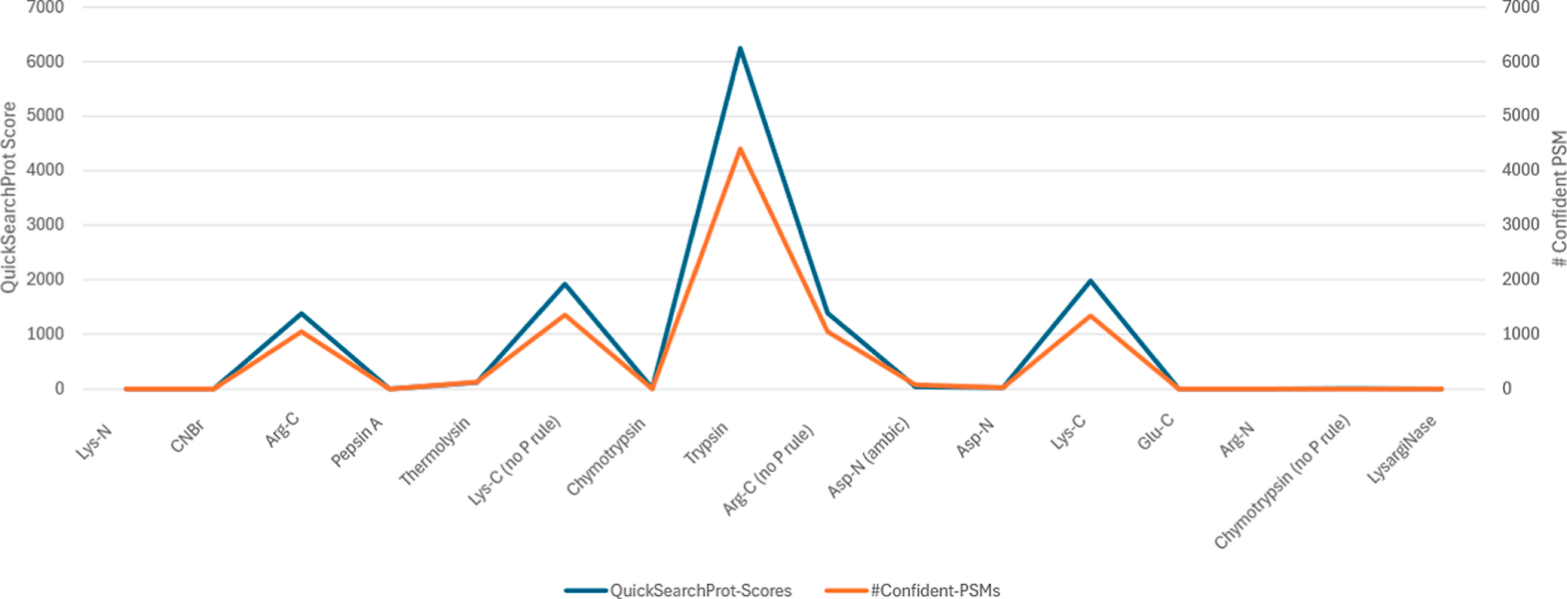
Comparison of CScores from QuickSearchProt and the number of confident PSMs resulted from searching data set 1 (PXD000674) with different enzyme values for Sage.

Although the CScore is based on the total number of identified spectra, it still performs better than using the number of identified spectra directly, as it adds the quality of the identified PSMs to the final score through the different weights, i.e., the ranks, for each PSM. This makes the CScore more sensitive when comparing the results from different searches. One example can be found in data set 1 (PXD000674), where including carbamidomethylation of C as a fixed modification in the X! Tandem search decreased the total number of identified PSMs by eight compared to the reference search. The CScore, however, improved to 41, indicating that the lost PSMs are of lower quality than those gained. This could be confirmed by searching the full data set without carbamidomethylation of C, which reduced the total number of identified PSMs from 5024 to 4901. Another example can be found in data set 2 (PXD000561), where the addition of deamidation of N as a variable modification with Sage decreased the number of identified PSMs by 27, while the CScore increased by 27. This result was also confirmed by comparing searches with and without deamidation of N, yielding results of 3353 and 3232 PSMs, respectively.

The number of validated spectra serves as a quick indicator of changes in the total number of single peptides without requiring peptide-to-protein mapping. This is particularly relevant for improving protein identifications. Based on calculated correlations, we observed a strong relationship between the total number of PSMs and single peptides, with a Pearson correlation coefficient of 0.996 across all comparisons using both Sage and X! Tandem search engines across six tested data sets.

This approach incorporates the intrinsic scoring behavior of each search engine, acknowledging that different algorithms may favor specific parameter values when assigning hyperscores. By leveraging validated identifications, the evaluation reflects not only the data set characteristics but also the algorithmic preferences of the search engine in use. This dual consideration enables a more nuanced and engine-aware performance metric, ensuring that the final results are shaped by both data-driven and algorithm-specific factors.

### Multiparameter Optimization and Order Effects

When multiple parameters are adjusted, it is computationally impractical to test all possible parameter combinations due to the exponential increase in the number of searches. Instead, each parameter is adjusted individually, with subsequent parameters adjusted based on the outcomes of the preceding ones. The order in which the parameters are tested is crucial for both accuracy and processing speed, as the process is sensitive to the initial parameter selections—if early parameters are suboptimal, this will negatively impact all subsequent selections. Furthermore, modifying the order of the parameters can lead to significantly different outcomes.

A comparison of the search parameters provided for the PRIDE projects, referred to as PRIDE mode, with those derived by QuickSearchProt in Subset mode revealed an agreement in most of the standard parameter values. As shown in Supporting Information Tables S4 and S5, the key differences lie in the PTM parameters. There were also noticeable differences in some of the advanced parameters for both X! Tandem and Sage, compared to the default values in SearchGUI.

Figure 6 demonstrates a clear improvement in the outcomes across all tested data sets. The intersection of identified PSMs (middle segment) shows that the results from the Subset mode cover most of the PSMs from the PRIDE mode while also adding many additional PSMs (lower segment). However, a small portion of the PSMs from the PRIDE mode results were not identified in the Subset mode (top segment). A comparison summary is available in Supporting Information Table S6.

**Figure 6:**
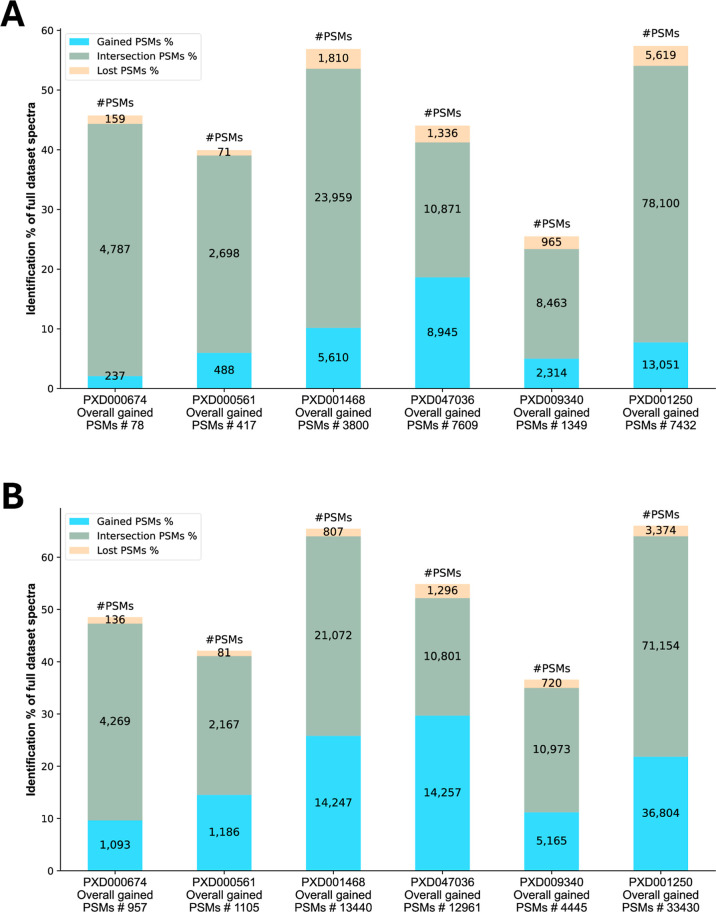
(A) Comparison of Subset mode vs PRIDE mode for X! Tandem. (B) Comparison of Subset mode vs PRIDE mode for Sage. The *y*-axis indicates the percentage of spectra identified from the complete spectrum file.

Evaluating the overall performance of multiple search parameters is inherently complex, as the effectiveness of each parameter relies not only on the selected value but also on the values of previously adjusted parameters. To tackle this challenge, the focus was moved from assessing the isolated effects of individual parameters into evaluating the combined impact of the resulting adjusted parameters. This was achieved by highlighting the intersection of identified spectra as well as the total gains (newly identified spectra) and losses (lost identified spectra) observed across the conducted searches. This comparative strategy is illustrated through comparisons of Subset mode versus Full Data mode (Figure 4) and Subset mode versus PRIDE mode (Figure 6).

To assess the overall performance, the results from the PRIDE mode were used as a reference point. The consistent improvement in the search results in Subset mode compared to PRIDE mode was evident across all data sets using both search engines, thus demonstrating that QuickSearchProt is able to consistently identify combinations of search parameter values that are equal to or superior to those provided in the PRIDE projects. It also highlights the benefits for inexperienced users or for situations where the available metadata is limited, demonstrating QuickSearchProt’s potential for reanalyzing public proteomics data sets, thereby enhancing data reusability and producing more robust analytical outcomes.

### Notable Data Set-Specific Differences

When comparing Subset mode versus Full Data mode, we observed notable variations that appear to stem from either the underlying characteristics of the data sets or the input reduction strategy. For example, in data set 3 (PXD001468) analyzed using Sage, the number of unique identifications in Full Data mode was nearly four times higher than in Subset mode, seemingly explained by differences in Sage’s advanced search parameters. Specifically, the peptide length range was set to 5–30 in Subset mode compared to 7–30 in Full Data mode, and the minimum fragment *m*/*z* threshold was 175 in Subset mode versus 150 in Full Data mode. This suggests that the subset configuration favored shorter peptides and applied a stricter fragment *m*/*z* threshold, likely due to a tag-picking strategy focused on short tags (length = 3), prioritizing confident, high-quality spectra. The elevated minimum fragment *m*/*z* in Subset mode may therefore be a deliberate choice to enhance the spectral quality by filtering out low-mass noise.

A second notable observation emerged when comparing results from data set 4 (PXD047036) using Subset mode versus PRIDE mode, using X! Tandem. QuickSearchProt’s adjusted search parameters here yielded 1.5 times more identifications. The difference was due to PRIDE indicating a precursor mass tolerance of 20 ppm and a fragment mass tolerance of 0.5 Da, while QuickSearchProt used 10 ppm and 0.02 Da, respectively. While the broader tolerances in PRIDE mode can facilitate the detection of spectra with unknown PTMs, they also tend to produce more low-confidence PSMs. This results in inflated identification counts with reduced reliability and a higher rate of false positives, which are subsequently filtered out during target-decoy analysis. QuickSearchProt mitigated this issue by explicitly incorporating common PTMs (deamidation of N and Q, oxidation of M, and N-terminal acetylation). Combined with its stricter mass tolerances (fragment and precursor), this strategy led to a more confident and accurate set of identifications.

The final example is drawn from data set 5 (PXD009340), comparing Subset mode and PRIDE mode, both analyzed using Sage. While the two modes agreed on most search parameters, they diverged on the set of variable modifications. QuickSearchProt selected dimethylation of K, oxidation of M, and pyroglutamate formation from E and Q, whereas PRIDE indicated acetylation of protein N-termini and oxidation of M. Additionally, QuickSearchProt applied stricter thresholds for several advanced search parameters such as minimum fragment *m*/*z* and the maximum number of peaks per spectrum. These tighter filters helped to reduce false positives by narrowing the search space. At the same time, the inclusion of specific variable modifications enabled the identification of spectra that would otherwise be missed, ultimately improving the number of confident PSMs from 11,693 to 16,138.

### Current Constraints and Limitations

QuickSearchProt currently supports two search engines: X! Tandem and Sage. However, the system is designed for easy extendibility, allowing it to easily incorporate additional search engines that are currently integrated within the SearchGUI framework, e.g., MS Amanda,^16^ MS-GF+,^5^ Comet,^17^ Tide,^18^ and MetaMorpheus.^19^

Second, QuickSearchProt depends on hyperscores that are not consistently available across all proteomics search engines. A potential solution would be to adopt a unified scoring approach similar to that used in PeptideShaker, where scores from various search engines are adjusted into a single, comparable metric. By adaptation of this score to calculate CScores, QuickSearchProt could enhance its compatibility and ensure consistent evaluation across a wider range of search engines.

Finally, the algorithm can at the moment be used only as a standalone application. However, future development aims to integrate QuickSearchProt as a plugin within SearchGUI. QuickSearchProt is already compatible with SearchGUI and could thus be seamlessly integrated into its interface, aiding in parameter selection and enhancing the user experience.

## Conclusion

Finding the most suitable search parameter values can be time-consuming if done manually and becomes substantially more complicated when dealing with multiple search parameters. Our algorithm automates this task and thus enables users to quickly identify suitable search parameter values by considering not only the data set specifications but also the search engine algorithm used. By conducting multiple searches using a carefully selected subset of spectra, a reduced sequence database, and a strategic order of the search parameters, the QuickSearchProt algorithm can identify suitable search parameter values without the need for expert knowledge, thus greatly simplifying the process of arriving at a set of suitable search parameters in any context.

## Supplementary Material



## Notes

Table S1: comparison of QuickSearchProt output in Subset mode and Full Data mode for X! Tandem; Table S2: comparison of QuickSearchProt output in Subset mode and Full Data mode for Sage; Table S3: comparison of CScores from QuickSearchProt and the number of confident PSMs resulting from searching data set 1 (PXD000674) with different enzyme values for Sage; Table S4: comparison of QuickSearchProt output in Subset mode and PRIDE data mode for X! Tandem; Table S5: comparison of QuickSearchProt output in Subset mode and PRIDE data mode for Sage; Table S6: summary of the comparison of the tested data sets between Subset mode and Full Data mode; and Table S7: summary of the comparison of the tested data sets between Subset mode and PRIDE mode (PDF)
