# Supplementary material for: Automatic Selection of Search Parameter Values for Mass Spectrometry-Based Search Engines
Source: J Proteome Res. 2026 Jan 24;25(2):662–71. doi: 10.1021/acs.jproteome.5c00641 (PMC12887993; doi:10.1021/acs.jproteome.5c00641)
Supplement: Supplementary file 1 [file pr5c00641_si_001.pdf]

Automatic selection of search parameter values for mass spectrometry-based search engines

Yehia M. Farag[1,2], Henrik Ø. Søgaard[1,2], Harald Barsnes[1,2,*]

[1] Proteomics Unit, Department of Biomedicine, University of Bergen, 5020 Bergen, Norway.

[2] Computational Biology Unit, Department of Informatics, University of Bergen, 5008 Bergen, Norway.

* Corresponding author. Email: harald.barsnes@uib.no

Table of contents

Table S1: Comparison of QuickSearchProt output in Subset Mode and Full Data Mode for X! Tandem.

Table S2: Comparison of QuickSearchProt output in Subset Mode and Full Data Mode for Sage.

Table S3: Comparison of CScores from QuickSearchProt and the number of confident PSMs resulting from searching Dataset 1 (PXD000674) with different enzyme values for Sage.

Table S4: Comparison of QuickSearchProt output in Subset Mode and PRIDE Data Mode for X! Tandem.

Table S5: Comparison of QuickSearchProt output in Subset Mode and PRIDE Data Mode for Sage.

Table S6: Summary of the comparison of the tested datasets between Subset Mode and Full Data Mode.

Table S7: Summary of the comparison of the tested datasets between Subset Mode and PRIDE Mode.

Supplementary Table 1: Comparison of QuickSearchProt output in Subset Mode and Full Data Mode for X! Tandem.

Dataset 1 - PXD000674 (41MB) #11332 (Sub-Dataset#: 1502)		
	**Sub-Dataset**	**Full-Dataset**
Subset init time	0:00:29	0:00:00
Params picking time	0:02:25	0:38:12
**Parameters**		
Digestion	Enzyme	Enzyme
Enzyme	Trypsin	Trypsin
Specificity	Specific	Specific
Max missed cleavages	2	0.02
Fragment ion types	B,Y	B,Y
Precursor accuracy (PPM)	10	10
Fragment accuracy (Da)	0.02	0.02
Precursor charge	2 to 4	2 to 4
Isotops	0 to 1	0 to 1
Fixed modifications	Carbamidomethylation of C	Carbamidomethylation of C
Variable modifications	Acetylation of peptide N-term<br>Deamidation of N<br>Methylation of K<br>Oxidation of M	Deamidation of N<br>Dimethylation of K<br>Oxidation of M
Spectrum dynamic range	100	100
Number of peaks	100	100
Minimum fragment (Mz)	100	100
Minimum peaks	5	5
Use noise suppression	No	No
Use parent isotop exp	Yes	Yes
Use quick acetyl	Yes	Yes
Use quick pyrolidone	Yes	Yes
Use stP bias	No	No
Use refinement stage	Yes	Yes
Unanticipated cleavage	Yes	Yes
Simi-enzymatic cleavage	Yes	Yes
Potintial modification	No	No
Use point mutations	No	Yes
Use SnAPs	Yes	Yes
Spectrum synthesis	Yes	Yes
Refined fixed modifications	Carbamidomethylation of C	Carbamidomethylation of C
Refined variable modifications		
Processing time	0:03:00	0:01:40
**# Identified proteins (PeptideShaker)**	1174	1172
**# Confident proteins (PeptideShaker)**	600	595
**# Unique peptides (PeptideShaker)**	3182	3211
**# Peptides (PeptideShaker)**	3454	3496
**# Identified spectra (PeptideShaker)**	**5024**	**5107**
**# Identified spectra (X!Tandem)**	4692	4748
**# Shared PSM**	**4945**	
**Parameter mismatch count**	**2**	

Orange color highlights the mismatched parameters

(*) The parameter is set to "No" for search testing because of its effect on the searching time.

Confident proteins, peptides, and PSMs are calculated based on PeptideShaker analysis results

Detailed documentation for each search parameter is available at www.thegpm.org/TANDEM/api/

Dataset 2 - PXD000561 (18.3MB) #8156 (Sub-Dataset: 1502)		
	**Sub-Dataset**	**Full-Dataset**
Subset init time	0:00:20	0:00:00
Params picking time	0:01:55	0:27:34
**Parameters**		
Digestion	Enzyme	Enzyme
Enzyme	Trypsin	Trypsin
Specificity	Specific	Specific
Max missed cleavages	2	2
Fragment ion types	B,Y	B,Y
Precursor accuracy (PPM)	10	10
Fragment accuracy (Da)	0.02	0.02
Precursor charge	2 to 4	2 to 4
Isotops	0 to 1	0 to 1
Fixed modifications	Carbamidomethylation of C	Carbamidomethylation of C
Variable modifications	Acetylation of peptide N-term<br>Deamidation of N<br>Dimethylation of K<br>Oxidation of M	Acetylation of peptide N-term<br>Deamidation of N<br>Dimethylation of K<br>Oxidation of M
Spectrum dynamic range	100	100
Number of peaks	100	100
Minimum fragment (Mz)	200	100
Minimum peaks	5	5
Use noise suppression	No	No
Use parent isotop exp	Yes	Yes
Use quick acetyl	Yes	Yes
Use quick pyrolidone	Yes	Yes
Use stP bias	No	No
Use refinement stage	Yes	Yes
Unanticipated cleavage	Yes	Yes
Simi-enzymatic cleavage	Yes	Yes
Potintial modification	No	No
Use point mutations	Yes	Yes
Use SnAPs	Yes	Yes
Spectrum synthesis	Yes	Yes
Refined fixed modifications	Carbamidomethylation of C	Carbamidomethylation of C
Refined variable modifications		
Processing time	0:00:49	0:00:49
**# Identified proteins (PeptideShaker)**	376	377
**# Confident proteins (PeptideShaker)**	237	226
**# Unique peptides (PeptideShaker)**	1321	1321
**# Peptides (PeptideShaker)**	1577	1571
**# Identified spectra (PeptideShaker)**	**3186**	**3227**
**# Identified spectra (X!Tandem)**	2818	3054
**# Shared PSM**	**2698**	
**Parameter mismatch count**	**1**	

Orange color highlights the mismatched parameters

(*) The parameter is set to "No" for search testing because of its effect on the searching time.

Confident proteins, peptides, and PSMs are calculated based on PeptideShaker analysis results

Detailed documentation for each search parameter is available at www.thegpm.org/TANDEM/api/

Dataset 3 - PXD001468 (222MB) #55188 (Sub-Dataset: 1502)		
	**Sub-Dataset**	**Full-Dataset**
Subset init time	0:01:05	0:00:00
Params picking time	0:04:06	4:31:29
**Parameters**		
Digestion	Enzyme	Enzyme
Enzyme	Trypsin	Trypsin
Specificity	Specific	Specific
Max missed cleavages	2	2
Fragment ion types	B,Y	B,Y
Precursor accuracy (PPM)	10	10
Fragment accuracy (Da)	0.02	0.02
Precursor charge	2 to 4	2 to 5
Isotops	0 to 1	0 to 1
Fixed modifications	Carbamidomethylation of C	Carbamidomethylation of C
Variable modifications	Acetylation of peptide N-term<br>Deamidation of N<br>Deamidation of Q<br>Oxidation of M	Acetylation of peptide N-term<br>Deamidation of N<br>Deamidation of Q<br>Oxidation of M
Spectrum dynamic range	100	100
Number of peaks	100	100
Minimum fragment (Mz)	100	100
Minimum peaks	5	5
Use noise suppression	No	No
Use parent isotop exp	Yes	Yes
Use quick acetyl	Yes	Yes
Use quick pyrolidone	Yes	Yes
Use stP bias	No	No
Use refinement stage	Yes	Yes
Unanticipated cleavage	Yes	Yes
Simi-enzymatic cleavage	Yes	Yes
Potintial modification	No	No
Use point mutations	No	No
Use SnAPs	Yes	Yes
Spectrum synthesis	Yes	Yes
Refined fixed modifications	Carbamidomethylation of C	Carbamidomethylation of C
Refined variable modifications		
Processing time	0:15:17	0:15:17
**# Identified proteins (PeptideShaker)**	4830	4830
**# Confident proteins (PeptideShaker)**	3209	3209
**# Unique peptides (PeptideShaker)**	16431	16431
**# Peptides (PeptideShaker)**	17421	17421
**# Identified spectra (PeptideShaker)**	**29569**	**29569**
**# Identified spectra (X!Tandem)**	24143	24143
**# Shared PSM**	**29569**	
**Parameter mismatch count**	**0**	

Orange color highlights the mismatched parameters

(*) The parameter is set to "No" for search testing because of its effect on the searching time.

Confident proteins, peptides, and PSMs are calculated based on PeptideShaker analysis results

Detailed documentation for each search parameter is available at www.thegpm.org/TANDEM/api/

Dataset 4 - PXD047036 (251MB)#48032 (Sub-Dataset: 1502)		
	**Sub-Dataset**	**Full-Dataset**
Subset init time	0:01:05	0:00:00
Params picking time	0:03:34	3:02:17
**Parameters**		
Digestion	Enzyme	Enzyme
Enzyme	Trypsin	Trypsin
Specificity	Specific	Specific
Max missed cleavages	2	2
Fragment ion types	B,Y	B,Y
Precursor accuracy (PPM)	10	10
Fragment accuracy (Da)	0.02	0.02
Precursor charge	2 to 4	2 to 5
Isotops	0 to 1	0 to 1
Fixed modifications	Carbamidomethylation of C	Carbamidomethylation of C
Variable modifications	Deamidation of N<br>Deamidation of Q<br>Oxidation of M	Acetylation of peptide N-term<br>Deamidation of N<br>Deamidation of Q<br>Oxidation of M
Spectrum dynamic range	100	100
Number of peaks	100	100
Minimum fragment (Mz)	150	100
Minimum peaks	5	5
Use noise suppression	No	No
Use parent isotop exp	Yes	Yes
Use quick acetyl	Yes	Yes
Use quick pyrolidone	Yes	Yes
Use stP bias	No	No
Use refinement stage	Yes	Yes
Unanticipated cleavage	Yes	Yes
Simi-enzymatic cleavage	Yes	Yes
Potintial modification	No	No
Use point mutations	Yes*	No*
Use SnAPs	Yes	Yes
Spectrum synthesis	Yes	Yes
Refined fixed modifications	Carbamidomethylation of C	Carbamidomethylation of C
Refined variable modifications		
Processing time	0:04:59	0:04:25
**# Identified proteins (PeptideShaker)**	1706	1631
**# Confident proteins (PeptideShaker)**	1227	1222
**# Unique peptides (PeptideShaker)**	10554	10688
**# Peptides (PeptideShaker)**	11241	11429
**# Identified spectra (PeptideShaker)**	**19816**	**20237**
**# Identified spectra (X!Tandem)**	18522	19248
**# Shared PSM**	**18656**	
**Parameter mismatch count**	**3**	

Orange color highlights the mismatched parameters

(*) The parameter is set to "No" for search testing because of its effect on the searching time.

Confident proteins, peptides, and PSMs are calculated based on PeptideShaker analysis results

Detailed documentation for each search parameter is available at www.thegpm.org/TANDEM/api/

Dataset 5 - PXD009340 (91.8MB) #46084 (Sub-Dataset: 1502)		
	**Sub-Dataset**	**Full-Dataset**
Sub-Dataset-Dataset init time	0:00:38	0:00:00
Params Picking time	0:03:46	1:55:50
**Parameters**		
Digestion	Enzyme	Enzyme
Enzyme	Trypsin	Trypsin
Specificity	Specific	Specific
Max missed cleavages	2	2
Fragment ion types	B,Y	B,Y
Precursor accuracy (PPM)	10	10
Fragment accuracy (Da)	0.02	0.02
Precursor charge	2 to 4	2 to 4
Isotops	0 to 1	0 to 1
Fixed modifications	Carbamidomethylation of C	Carbamidomethylation of C
Variable modifications	Deamidation of N<br>Methylation of K<br>Oxidation of M	Deamidation of N<br>Deamidation of Q<br>Oxidation of M
Spectrum dynamic range	180	180
Number of peaks	100	100
Minimum fragment (Mz)	100	100
Minimum peaks	5	5
Use noise suppression	No	No
Use parent isotop exp	Yes	Yes
Use quick acetyl	Yes	Yes
Use quick pyrolidone	Yes	Yes
Use stP bias	No	No
Use refinement stage	Yes	Yes
Unanticipated cleavage	Yes	Yes
Simi-enzymatic cleavage	Yes	Yes
Potintial modification	No	Yes*
Use SnAPs	Yes*	Yes*
Spectrum synthesis	Yes	Yes
Refined fixed modifications	Yes	Yes
Refined variable modifications	Carbamidomethylation of C	Carbamidomethylation of C
Processing time	0:02:44	0:01:24
**# Identified proteins (PeptideShaker)**	1672	1682
**# Confident proteins (PeptideShaker)**	1229	1271
**# Unique peptides (PeptideShaker)**	8186	8141
**# Peptides (PeptideShaker)**	9091	9033
**# Identified spectra (PeptideShaker)**	**10777**	**10776**
**# Identified spectra (X!Tandem)**	3908	3928
**# Shared PSM**	**10280**	
**Parameter mismatch count**	**2**	

Orange color highlights the mismatched parameters

(*) The parameter is set to "No" for search testing because of its effect on the searching time.

Confident proteins, peptides, and PSMs are calculated based on PeptideShaker analysis results

Detailed documentation for each search parameter is available at www.thegpm.org/TANDEM/api/

Dataset 6 - PXD001250 (672MB) #168634 (Sub-Dataset: 2002)		
	**Sub-Dataset**	**Full-Dataset**
Subset init time	0:02:48	0:00:00
Params picking time	0:06:00	26:27:23
**Parameters**		
Digestion	Enzyme	Enzyme
Enzyme	Trypsin	Trypsin
Specificity	Specific	Specific
Max missed cleavages	2	2
Fragment ion types	B,Y	B,Y
Precursor accuracy (PPM)	10	10
Fragment accuracy (Da)	0.02	0.02
Precursor charge	2 to 4	2 to 4
Isotops	0 to 1	0 to 1
Fixed modifications	Carbamidomethylation of C	Carbamidomethylation of C
Variable modifications	Deamidation of N<br>Deamidation of Q<br>Oxidation of M	Acetylation of peptide N-term<br>Deamidation of N<br>Deamidation of Q<br>Oxidation of M
Spectrum dynamic range	100	100
Number of peaks	100	100
Minimum fragment (Mz)	250	100
Minimum peaks	5	5
Use noise suppression	No	No
Use parent isotop exp	Yes	Yes
Use quick acetyl	Yes	Yes
Use quick pyrolidone	Yes	Yes
Use stP bias	No	No
Use refinement stage	Yes	Yes
Unanticipated cleavage	Yes	Yes
Simi-enzymatic cleavage	Yes	Yes
Potintial modification	No	No
Use point mutations	Yes*	Yes*
Use SnAPs	Yes	Yes
Spectrum synthesis	Yes	Yes
Refined fixed modifications	Carbamidomethylation of C	Carbamidomethylation of C
Refined variable modifications		
Processing time	0:38:00	00:65:23
**# Identified proteins (PeptideShaker)**	5607	5543
**# Confident proteins (PeptideShaker)**	5007	4966
**# Unique peptides (PeptideShaker)**	57980	57756
**# Peptides (PeptideShaker)**	61879	61779
**# Identified spectra (PeptideShaker)**	**91151**	**90653**
**# Identified spectra (X!Tandem)**	78328	83764
**# Shared PSM**	**85990**	
**Parameter mismatch count**	**2**	

Orange color highlights the mismatched parameters

(*) The parameter is set to "No" for search testing because of its effect on the searching time.

Confident proteins, peptides, and PSMs are calculated based on PeptideShaker analysis results

Detailed documentation for each search parameter is available at www.thegpm.org/TANDEM/api/

Supplementary Table 2: Comparison of QuickSearchProt output in Subset Mode and Full Data Mode for Sage.

Dataset 1 - PXD000674 (41MB) #11332 (Subset Mode#: 3001)		
	**Sub-Dataset**	**Full-Dataset**
Subset init time	0:02:02	00.00.00
Params picking time	0:02:05	0:45:00
**Parameters**		
Digestion	Enzyme	Enzyme
Enzyme	Trypsin	Trypsin
Specificity	Specific	Specific
Max missed cleavages	2	2
Fragment ion types	B,Y	B,Y
Precursor accuracy (PPM)	10	10
Fragment accuracy (Da)	0.02	0.02
Precursor charge	2 to 4	2 to 4
Isotops	0 to 1	0 to 1
Fixed modifications	Carbamidomethylation of C	Carbamidomethylation of C
Variable modifications	Deamidation of N<br>Dimethylation of K<br>Oxidation of M<br>Pyrolidone from Q	Deamidation of N<br>Dimethylation of K<br>Oxidation of M<br>Pyrolidone from Q
Peptide length (min-max)	7 to 30	6 to 30
Fragment Mz (min to max)	150 to 2000	150 to 2000
Peptide mass (min to max)	600.0 to 5000.0	600.0 to 5000.0
Min ion index	2	2
Maximum variable modifications	3	3
Generate decoy	Yes	Yes
Deisotope	Yes	Yes
Chimeric spectra	No	No
Predict retention time	Yes	Yes
Number of peaks (min to max)	15 to 180	15 to 190
Minimum mached peaks	3	3
Maximum fragment charge	1	1
Wide window	Yes*	No
Processing time	0:01:20	0:01:04
**# Identified proteins (PeptideShaker)**	1457	1286
**# Confident proteins (PeptideShaker)**	565	536
**# Unique peptides (PeptideShaker)**	3671	3676
**# Peptides (PeptideShaker)**	3936	3939
**# Identified spectra (PeptideShaker)**	**5362**	**5204**
**# Shared PSM**	**5173**	
**Parameter mismatch count**	**3**	

Orange color highlights the mismatched parameters

(*) The parameter is set to "No" for search testing because of its effect on the searching time.

Confident proteins, peptides, and PSMs are calculated based on PeptideShaker analysis results

Detailed documentation for each search parameter is available at www.sage-docs.vercel.app/docs

Dataset 2 - PXD000561 (18.3MB) #8156 (Subset Mode: 3000)		
	**Subset Mode**	**Full Data Mode**
Subset init time	0:00:33	0:00:46
Params picking time	0:02:40	0:34:12
**Parameters**		
Digestion	Enzyme	Enzyme
Enzyme	Trypsin	Trypsin
Specificity	Specific	Specific
Max missed cleavages	2	2
Fragment ion types	B,Y	B,Y
Precursor accuracy (PPM)	10	10
Fragment accuracy (Da)	0.02	0.02
Precursor charge	2 to 4	2 to 4
Isotops	0 to 1	0 to 1
Fixed modifications	Carbamidomethylation of C	Carbamidomethylation of C
Variable modifications	Deamidation of N<br>Methylation of K<br>Oxidation of M<br>Pyrolidone from Q	Deamidation of N<br>Methylation of K<br>Oxidation of M<br>Pyrolidone from Q
Peptide length (min-max)	7 to 30	6 to 30
Fragment Mz (min to max)	150 to 2000	150 to 1750
Peptide mass (min to max)	600.0 to 5000.0	600.0 to 5000.0
Min ion index	2	3
Maximum variable modifications	3	3
Generate decoy	Yes	Yes
Deisotope	Yes	Yes
Chimeric spectra	No	No
Predict retention time	Yes	Yes
Number of peaks (min to max)	10 to 100	10 to 190
Minimum mached peaks	3	3
Maximum fragment charge	1	1
Wide window	No	No
Processing time	0:00:40	0:00:11
**# Identified proteins (PeptideShaker)**	570	495
**# Confident proteins (PeptideShaker)**	221	197
**# Unique peptides (PeptideShaker)**	1334	1280
**# Peptides (PeptideShaker)**	1526	1476
**# Identified spectra (PeptideShaker)**	**3353**	**3408**
**# Shared PSM**	**3132**	
**Parameter mismatch count**	**4**	

Orange color highlights the mismatched parameters

(*) The parameter is set to "No" for search testing because of its effect on the searching time.

Confident proteins, peptides, and PSMs are calculated based on PeptideShaker analysis results

Detailed documentation for each search parameter is available at www.sage-docs.vercel.app/docs

Dataset 3 - PXD001468 (222MB) #55188 (Subset Mode: 3002)		
	**Subset Mode**	**Full Data Mode**
Subset init time	0:01:34	00.00.00
Params picking time	0:06:03	0:50:22
**Parameters**		
Digestion	Enzyme	Enzyme
Enzyme	Trypsin	Trypsin
Specificity	Specific	Specific
Max missed cleavages	2	2
Fragment ion types	B,Y	B,Y
Precursor accuracy (PPM)	10	10
Fragment accuracy (Da)	0.02	0.02
Precursor charge	2 to 4	2 to 4
Isotops	0 to 1	0 to 1
Fixed modifications	Carbamidomethylation of C	Carbamidomethylation of C
Variable modifications	Deamidation of N<br>Deamidation of Q<br>Oxidation of M<br>Pyrolidone from Q	Deamidation of N<br>Deamidation of Q<br>Oxidation of M<br>Pyrolidone from Q
Peptide length (min-max)	5 to 30	7 to 30
Fragment Mz (min to max)	175 to 2000	150 to 2000
Peptide mass (min to max)	600.0 to 5000.0	600.0 to 5000.0
Min ion index	2	0
Maximum variable modifications	3	3
Generate decoy	Yes	Yes
Deisotope	Yes	Yes
Chimeric spectra	No	No
Predict retention time	Yes	Yes
Number of peaks (min to max)	15 to 200	15 to 200
Minimum mached peaks	4	4
Maximum fragment charge	1	1
Wide window	Yes*	No
Processing time	0:00:59	0:01:12
**# Identified proteins (PeptideShaker)**	6803	6274
**# Confident proteins (PeptideShaker)**	3538	3395
**# Unique peptides (PeptideShaker)**	17860	17353
**# Peptides (PeptideShaker)**	18756	18236
**# Identified spectra (PeptideShaker)**	**35319**	**31160**
**# Shared PSM**	**29917**	
**Parameter mismatch count**	**4**	

Orange color highlights the mismatched parameters

(*) The parameter is set to "No" for search testing because of its effect on the searching time.

Confident proteins, peptides, and PSMs are calculated based on PeptideShaker analysis results

Detailed documentation for each search parameter is available at www.sage-docs.vercel.app/docs

Dataset 4 - PXD047036 (251MB) #48032 (Subset Mode: 3002)		
	**Subset Mode**	**Full Data Mode**
Subset init time	0:01:31	00.00.00
Params picking time	0:02:56	0:44:11
**Parameters**		
Digestion	Enzyme	Enzyme
Enzyme	Trypsin	Trypsin
Specificity	Specific	Specific
Max missed cleavages	2	2
Fragment ion types	B,Y	B,Y
Precursor accuracy (PPM)	10	10
Fragment accuracy (Da)	0.02	0.02
Precursor charge	2 to 4	2 to 4
Isotops	0 to 1	0 to 1
Fixed modifications	Carbamidomethylation of C	Carbamidomethylation of C
Variable modifications	Deamidation of N<br>Deamidation of Q<br>Oxidation of M<br>Pyrolidone from Q	Deamidation of N<br>Deamidation of Q<br>Oxidation of M<br>Pyrolidone from Q
Peptide length (min-max)	7 to 30	6 to 30
Fragment Mz (min to max)	150 to 2000	150 to 1750
Peptide mass (min to max)	600.0 to 5000.0	600.0 to 5000.0
Min ion index	2	4
Maximum variable modifications	3	3
Generate decoy	Yes	Yes
Deisotope	Yes	Yes
Chimeric spectra	No	No
Predict retention time	Yes	Yes
Number of peaks (min to max)	15 to 180.	15 to 200
Minimum mached peaks	3	4
Maximum fragment charge	1	1
Wide window	Yes*	Yes*
Processing time	0:01:02	0:00:57
**# Identified proteins (PeptideSha**	3910	3905
**# Confident proteins (PeptideSha**	1606	1836
**# Unique peptides (PeptideShak**	13543	14232
**# Peptides (PeptideShaker)**	14349	15017
**# Identified spectra (PeptideShak**	**25058**	**25101**
**# Shared PSM**	**22442**	
**Parameter mismatch count**	**5**	

Orange color highlights the mismatched parameters

(*) The parameter is set to "No" for search testing because of its effect on the searching time.

Confident proteins, peptides, and PSMs are calculated based on PeptideShaker analysis results

Detailed documentation for each search parameter is available at www.sage-docs.vercel.app/docs

Dataset 5 - PXD009340(91.8MB) #46084 (Subset Mode: 3002)		
	**Subset Mode**	**Full Data Mode**
Subset init time	0:00:55	00.00.00
Params picking time	00:06:05	0:33:59
**Parameters**		
Digestion	Enzyme	Enzyme
Enzyme	Trypsin	Trypsin
Specificity	Specific	Specific
Max missed cleavages	2	2
Fragment ion types	B,Y	B,Y
Precursor accuracy (PPM)	10	10
Fragment accuracy (Da)	0.02	0.02
Precursor charge	2 to 4	2 to 4
Isotops	0 to 1	0 to 1
Fixed modifications	Carbamidomethylation of C	Carbamidomethylation of C
Variable modifications	Dimethylation of K<br>Oxidation of M<br>Pyrolidone from E<br>Pyrolidone from Q	Acetylation of protein N-term<br>Oxidation of M<br>Pyrolidone from E<br>Pyrolidone from Q
Peptide length (min-max)	8 to 30	8 to 26.
Fragment Mz (min to max)	150 to 2000	200 to 1500
Peptide mass (min to max)	600.0 to 5000.0	600.0 to 5000.0
Min ion index	2	2
Maximum variable modifications	2	1
Generate decoy	Yes	Yes
Deisotope	No	No
Chimeric spectra	No	No
Predict retention time	Yes	Yes
Number of peaks (min to max)	15 to 120	15 to 150
Minimum mached peaks	3	3
Maximum fragment charge	1	1
Wide window	No	No
Processing time	0:00:13	0:00:16
**# Identified proteins (PeptideShaker)**	3274	3071
**# Confident proteins (PeptideShaker)**	1610	1659
**# Unique peptides (PeptideShaker)**	12002	11736
**# Peptides (PeptideShaker)**	12804	12537
**# Identified spectra (PeptideShaker)**	**16138**	**15759**
**# Shared PSM**	**12204**	
**Parameter mismatch count**	**5**	

Orange color highlights the mismatched parameters

(*) The parameter is set to "No" for search testing because of its effect on the searching time.

Confident proteins, peptides, and PSMs are calculated based on PeptideShaker analysis results

Detailed documentation for each search parameter is available at www.sage-docs.vercel.app/docs

Dataset 6 - PXD001250 (672MB) #168634 (Subset Mode: 3002)		
	**Subset Mode**	**Full Data Mode**
Subset init time	0:03:16	00.00.00
Params picking time	0:04:18	1:16:21
**Parameters**		
Digestion	Enzyme	Enzyme
Enzyme	Trypsin	Trypsin
Specificity	Specific	Specific
Max missed cleavages	2	2
Fragment ion types	B,Y	B,Y
Precursor accuracy (PPM)	10	10
Fragment accuracy (Da)	0.02	0.02
Precursor charge	2 to 4	2 to 4
Isotops	0 to 1	0 to 1
Fixed modifications	Carbamidomethylation of C	Carbamidomethylation of C
Variable modifications	Deamidation of N<br>Deamidation of Q<br>Oxidation of M<br>Pyrolidone from Q	Deamidation of N<br>Deamidation of Q<br>Oxidation of M<br>Pyrolidone from Q
Peptide length (min-max)	7 to 30	7 to 30
Fragment Mz (min to max)	175 to 1750	150 to 2000
Peptide mass (min to max)	600.0 to 5000.0	600.0 to 5000.0
Min ion index	2	2
Maximum variable modifications	3	3
Generate decoy	Yes	Yes
Deisotope	Yes	Yes
Chimeric spectra	No	No
Predict retention time	Yes	Yes
Number of peaks (min to max)	15 to 200	15 to 200
Minimum mached peaks	4	4
Maximum fragment charge	1	1
Wide window	Yes*	Yes*
Processing time	0:00:56	0:00:39
**# Identified proteins (PeptideShaker)**	9889	9009
**# Confident proteins (PeptideShaker)**	7986	5929
**# Unique peptides (PeptideShaker)**	74965	69016
**# Peptides (PeptideShaker)**	78985	72945
**# Identified spectra (PeptideShaker)**	**107958**	**104783**
**# Shared PSM**	**100636**	
**Parameter mismatch count**	**1**	

Orange color highlights the mismatched parameters

(*) The parameter is set to "No" for search testing because of its effect on the searching time.

Confident proteins, peptides, and PSMs are calculated based on PeptideShaker analysis results

Detailed documentation for each search parameter is available at www.sage-docs.vercel.app/docs

Supplementary Table 3: Comparison of CScores from QuickSearchProt and the number of confident PSMs resulting from searching Dataset 1 (PXD000674) with different enzyme values for Sage.

Enzyme	CScores	#PSMs	FULL Data time	#Confident-PSMs
Lys-N	4	1	00:00:23	0
CNBr	0	0	00:00:06	0
Arg-C	1384	346	00:00:20	1050
Pepsin A	0	0	0:01:53	2
Thermolysin	120	30	0:01:25	129
Lys-C (no P rule)	1920	480	00:00:31	1358
Chymotrypsin	8	2	00:02:19	4
Trypsin	**6244**	**1561**	00:01:35	4405
Arg-C (no P rule)	1388	347	00:00:31	1051
Asp-N (ambic)	40	10	00:02:32	73
Asp-N	28	7	00:00:16	23
Lys-C	1980	495	00:00:21	1344
Glu-C	0	0	00:00:31	1
Arg-N	4	1	00:00:20	3
Chymotrypsin (no P rule)	8	2	00:03:00	0
LysargiNase	0	0	00:01:52	0
**Total time**	**0:00:39**		**00:12:05**	

*** The colors in the table represent the values in each cell, where darker shades within a column indicate higher values, making it easier to visualize differences in scores.**

Supplementary Table 4: Comparison of QuickSearchProt output in Subset Mode and PRIDE Data Mode for X! Tandem.

Dataset 1 - PXD000674 (41MB) #11332 (Sub-Dataset#: 1502)		
	**Sub-Dataset**	**PRIDE**
Subset init time	0:00:29	0:00:00
Params picking time	0:02:25	0:00:00
**Parameters**		
Digestion	Enzyme	Enzyme
Enzyme	Trypsin	Trypsin
Specificity	Specific	Specific
Max missed cleavages	2	2
Fragment ion types	B,Y	B,Y
Precursor accuracy (PPM)	10	10
Fragment accuracy (Da)	0.02	0.02
Precursor charge	2 to 4	2 to 4
Isotops	0 to 1	0 to 1
Fixed modifications	Carbamidomethylation of C	Carbamidomethylation of C
Variable modifications	Acetylation of peptide N-term<br>Deamidation of N<br>Methylation of K<br>Oxidation of M	Oxidation of M<br>Phosphorylation of Ser/Thr/Tyr
Spectrum dynamic range	100	100
Number of peaks	100	50
Minimum fragment (Mz)	100	200
Minimum peaks	5	5
Use noise suppression	No	No
Use parent isotop exp	Yes	Yes
Use quick acetyl	Yes	Yes
Use quick pyrolidone	Yes	Yes
Use stP bias	No	No
Use refinement stage	Yes	Yes
Unanticipated cleavage	Yes	Yes
Simi-enzymatic cleavage	Yes	No
Potintial modification	No	No
Use point mutations	No	No
Use SnAPs	Yes	Yes
Spectrum synthesis	Yes	Yes
Refined fixed modifications	Carbamidomethylation of C	Carbamidomethylation of C
Refined variable modifications		
Processing time	0:03:00	0:00:54
**# Identified proteins (PeptideShaker)**	1174	1192
**# Confident proteins (PeptideShaker)**	600	593
**# Unique peptides (PeptideShaker)**	3182	3141
**# Peptides (PeptideShaker)**	3454	3401
**# Identified spectra (PeptideShaker)**	**5024**	**4946**
**# Identified spectra (X!Tandem)**	4692	4379
**# Shared PSM**	**4787**	

Orange color highlights the mismatched parameters

(*) The parameter is set to "No" for search testing because of its effect on the searching time.

Confident proteins, peptides, and PSMs are calculated based on PeptideShaker analysis results

Detailed documentation for each search parameter is available at www.thegpm.org/TANDEM/api/

Dataset 2 - PXD000561 (18.3MB) #8156 (Sub-Dataset: 1502)		
	**Sub-Dataset**	**PRIDE**
Subset init time	0:00:20	0:00:00
Params picking time	0:01:55	0:35:49
**Parameters**		
Digestion	Enzyme	Enzyme
Enzyme	Trypsin	Trypsin
Specificity	Specific	Specific
Max missed cleavages	2	1
Fragment ion types	B,Y	B,Y
Precursor accuracy (PPM)	10	10
Fragment accuracy (Da)	0.02	0.05
Precursor charge	2 to 4	2 to 4
Isotops	0 to 1	0 to 1
Fixed modifications	Carbamidomethylation of C	Carbamidomethylation of C
Variable modifications	Acetylation of peptide N-term<br>Deamidation of N<br>Dimethylation of K<br>Oxidation of M	Oxidation of M<br>Acetylation of protein N-termini<br>PyroQ
Spectrum dynamic range	100	100
Number of peaks	100	50
Minimum fragment (Mz)	200	200
Minimum peaks	5	5
Use noise suppression	No	No
Use parent isotop exp	Yes	Yes
Use quick acetyl	Yes	Yes
Use quick pyrolidone	Yes	Yes
Use stP bias	No	No
Use refinement stage	Yes	Yes
Unanticipated cleavage	Yes	Yes
Simi-enzymatic cleavage	Yes	No
Potintial modification	No	No
Use point mutations	Yes	No
Use SnAPs	Yes	Yes
Spectrum synthesis	Yes	Yes
Refined fixed modifications	Carbamidomethylation of C	Carbamidomethylation of C
Refined variable modifications		
Processing time	0:00:49	0:01:06
**# Identified proteins (PeptideShaker)**	376	357
**# Confident proteins (PeptideShaker)**	237	219
**# Unique peptides (PeptideShaker)**	1321	1059
**# Peptides (PeptideShaker)**	1577	1288
**# Identified spectra (PeptideShaker)**	**3186**	**2769**
**# Identified spectra (X!Tandem)**	2818	2162
**# Shared PSM**	**2698**	

Orange color highlights the mismatched parameters

(*) The parameter is set to "No" for search testing because of its effect on the searching time.

Confident proteins, peptides, and PSMs are calculated based on PeptideShaker analysis results

Detailed documentation for each search parameter is available at www.thegpm.org/TANDEM/api/

Dataset 3 - PXD001468 (222MB) #55188 (Sub-Dataset: 1502)		
	**Sub-Dataset**	**PRIDE**
Subset init time	0:01:05	0:00:00
Params picking time	0:04:06	00.00.00
**Parameters**		
Digestion	Enzyme	Enzyme
Enzyme	Trypsin	Trypsin
Specificity	Specific	Specific
Max missed cleavages	2	1
Fragment ion types	B,Y	B,Y
Precursor accuracy (PPM)	10	5
Fragment accuracy (Da)	0.02	0.02
Precursor charge	2 to 4	2 to 4
Isotops	0 to 1	0 to 1
Fixed modifications	Carbamidomethylation of C	Carbamidomethylation of C
Variable modifications	Acetylation of peptide N-term<br>Deamidation of N<br>Deamidation of Q<br>Oxidation of M	Oxidation of M
Spectrum dynamic range	100	100
Number of peaks	100	50
Minimum fragment (Mz)	100	200
Minimum peaks	5	5
Use noise suppression	No	No
Use parent isotop exp	Yes	Yes
Use quick acetyl	Yes	Yes
Use quick pyrolidone	Yes	Yes
Use stP bias	No	No
Use refinement stage	Yes	Yes
Unanticipated cleavage	Yes	Yes
Simi-enzymatic cleavage	Yes	No
Potintial modification	No	No
Use point mutations	No	No
Use SnAPs	Yes	Yes
Spectrum synthesis	Yes	Yes
Refined fixed modifications	Carbamidomethylation of C	Carbamidomethylation of C
Refined variable modifications		
Processing time	0:15:17	0:03:34
**# Identified proteins (PeptideShaker)**	4830	4826
**# Confident proteins (PeptideShaker)**	3209	3102
**# Unique peptides (PeptideShaker)**	16431	14323
**# Peptides (PeptideShaker)**	17421	15127
**# Identified spectra (PeptideShaker)**	**29569**	**25769**
**# Identified spectra (X!Tandem)**	24143	19707
**# Shared PSM**	**23959**	

Orange color highlights the mismatched parameters

(*) The parameter is set to "No" for search testing because of its effect on the searching time.

Confident proteins, peptides, and PSMs are calculated based on PeptideShaker analysis results

Detailed documentation for each search parameter is available at www.thegpm.org/TANDEM/api/

Dataset 4 - PXD047036 (251MB) #48032 (Sub-Dataset: 1502)		
	**Sub-Dataset**	**PRIDE**
Subset init time	0:01:05	0:00:00
Params picking time	0:03:34	00.00.00
**Parameters**		
Digestion	Enzyme	Enzyme
Enzyme	Trypsin	Trypsin
Specificity	Specific	Specific
Max missed cleavages	2	2
Fragment ion types	B,Y	B,Y
Precursor accuracy (PPM)	10	20
Fragment accuracy (Da)	0.02	0.5
Precursor charge	2 to 4	2 to 4
Isotops	0 to 1	0 to 1
Fixed modifications	Carbamidomethylation of C	Carbamidomethylation of C
Variable modifications	Deamidation of N<br>Deamidation of Q<br>Oxidation of M	Acetylation of protein N-termini<br>Oxidation of M
Spectrum dynamic range	100	100
Number of peaks	100	50
Minimum fragment (Mz)	150	200
Minimum peaks	5	5
Use noise suppression	No	No
Use parent isotop exp	Yes	Yes
Use quick acetyl	Yes	Yes
Use quick pyrolidone	Yes	Yes
Use stP bias	No	No
Use refinement stage	Yes	Yes
Unanticipated cleavage	Yes	Yes
Simi-enzymatic cleavage	Yes	No
Potintial modification	No	No
Use point mutations	Yes*	No
Use SnAPs	Yes	Yes
Spectrum synthesis	Yes	Yes
Refined fixed modifications	Carbamidomethylation of C	Carbamidomethylation of C
Refined variable modifications		
Processing time	0:04:59	0:01:53
**# Identified proteins (PeptideShaker)**	1706	1386
**# Confident proteins (PeptideShaker)**	1227	943
**# Unique peptides (PeptideShaker)**	10554	6159
**# Peptides (PeptideShaker)**	11241	6540
**# Identified spectra (PeptideShaker)**	**19816**	**12207**
**# Identified spectra (X!Tandem)**	18522	11663
**# Shared PSM**	**10871**	

Orange color highlights the mismatched parameters

(*) The parameter is set to "No" for search testing because of its effect on the searching time.

Confident proteins, peptides, and PSMs are calculated based on PeptideShaker analysis results

Detailed documentation for each search parameter is available at www.thegpm.org/TANDEM/api/

Dataset 5 - PXD009340(91.8MB) #46084 (Sub-Dataset: 1502)		
	**Subset-mode**	**PRIDE**
Subset init time	0:00:38	0:00:00
Params picking time	0:03:46	00.00.00
**Parameters**		
Digestion	Enzyme	Enzyme
Enzyme	Trypsin	Trypsin
Specificity	Specific	Specific
Max missed cleavages	2	2
Fragment ion types	B,Y	B,Y
Precursor accuracy (PPM)	10	10
Fragment accuracy (Da)	0.02	0.02
Precursor charge	2 to 4	2 to 4
Isotops	0 to 1	0 to 1
Fixed modifications	Carbamidomethylation of C	Carbamidomethylation of C
Variable modifications	Deamidation of N<br>Methylation of K<br>Oxidation of M	Acetylation of protein N-termini Oxidation of M
Spectrum dynamic range	180	100
Number of peaks	100	50
Minimum fragment (Mz)	100	200
Minimum peaks	5	5
Use noise suppression	No	No
Use parent isotop exp	Yes	Yes
Use quick acetyl	Yes	Yes
Use quick pyrolidone	Yes	Yes
Use stP bias	No	No
Use refinement stage	Yes	Yes
Unanticipated cleavage	Yes	Yes
Simi-enzymatic cleavage	Yes	No
Potintial modification	No	No
Use point mutations	Yes*	No
Use SnAPs	Yes	Yes
Spectrum synthesis	Yes	Yes
Refined fixed modifications	Carbamidomethylation of C	Carbamidomethylation of C
Refined variable modifications		
Processing time	0:02:44	0:01:02
**# Identified proteins (PeptideS**	1672	1659
**# Confident proteins (Peptide**	1229	1232
**# Unique peptides (PeptideSh**	8186	7347
**# Peptides (PeptideShaker)**	9091	8128
**# Identified spectra (PeptideSh**	**10777**	**9428**
**# Identified spectra (X!Tanden**	3908	2164
**# Shared PSM**	**8463**	

Orange color highlights the mismatched parameters

(*) The parameter is set to "No" for search testing because of its effect on the searching time.

Confident proteins, peptides, and PSMs are calculated based on PeptideShaker analysis results

Detailed documentation for each search parameter is available at www.thegpm.org/TANDEM/api/

Dataset 6 - PXD001250 (672MB) #168634 (Sub-Dataset: 2002)		
	**Subset-mode**	**PRIDE**
Subset init time	0:02:48	0:00:00
Params picking time	0:06:00	00.00.00
**Parameters**		
Digestion	Enzyme	Enzyme
Enzyme	Trypsin	Trypsin
Specificity	Specific	Specific
Max missed cleavages	2	2
Fragment ion types	B,Y	B,Y
Precursor accuracy (PPM)	10	10
Fragment accuracy (Da)	0.02	0.02
Precursor charge	2 to 4	2 to 4
Isotops	0 to 1	0 to 1
Fixed modifications	Carbamidomethylation of C	Carbamidomethylation of C
Variable modifications	Deamidation of N<br>Deamidation of Q<br>Oxidation of M	Acetylation of protein N-termini<br>Oxidation of M
Spectrum dynamic range	100	100
Number of peaks	100	50
Minimum fragment (Mz)	250	200
Minimum peaks	5	5
Use noise suppression	No	No
Use parent isotop exp	Yes	Yes
Use quick acetyl	Yes	Yes
Use quick pyrolidone	Yes	Yes
Use stP bias	No	No
Use refinement stage	Yes	Yes
Unanticipated cleavage	Yes	Yes
Simi-enzymatic cleavage	Yes	No
Potintial modification	No	No
Use point mutations	Yes*	No
Use SnAPs	Yes	Yes
Spectrum synthesis	Yes	Yes
Refined fixed modifications	Carbamidomethylation of C	Carbamidomethylation of C
Refined variable modifications		
Processing time	0:38:00	0:21:45
**# Identified proteins (PeptideShaker)**	5607	5512
**# Confident proteins (PeptideShaker)**	5007	4890
**# Unique peptides (PeptideShaker)**	57980	53113
**# Peptides (PeptideShaker)**	61879	56703
**# Identified spectra (PeptideShaker)**	**91151**	**83719**
**# Identified spectra (X!Tandem)**	78328	74109
**# Shared PSM**	**78100**	

Orange color highlights the mismatched parameters

(*) The parameter is set to "No" for search testing because of its effect on the searching time.

Confident proteins, peptides, and PSMs are calculated based on PeptideShaker analysis results

Detailed documentation for each search parameter is available at www.thegpm.org/TANDEM/api/

Supplementary Table 5: Comparison of QuickSearchProt output in Subset Mode and PRIDE Data Mode for Sage.

Dataset 1 - PXD000674 (41MB) #11332 (Subset Mode#: 3001)		
	**Subset Mode**	**PRIDE Mode**
Subset init time	0:02:02	00.00.00
Params picking time	0:02:05	00.00.00
**Parameters**		
Digestion	Enzyme	Enzyme
Enzyme	Trypsin	Trypsin
Specificity	Specific	Specific
Max missed cleavages	2	2
Fragment ion types	B,Y	B,Y
Precursor accuracy (PPM)	10	10
Fragment accuracy (Da)	0.02	0.02
Precursor charge	2 to 4	2 to 4
Isotops	0 to 1	0 to 1
Fixed modifications	Carbamidomethylation of C	Carbamidomethylation of C
Variable modifications	Deamidation of N<br>Dimethylation of K<br>Oxidation of M<br>Pyrolidone from Q	Oxidation of M<br>Phosphorylation of Ser/Thr/Tyr
Peptide length (min-max)	7 to 30	8 to 30
Fragment Mz (min to max)	150 to 2000	200 to 2000
Peptide mass (min to max)	600.0 to 5000.0	600.0 to 5000.0
Min ion index	2	2
Maximum variable modifications	3	2
Generate decoy	Yes	No
Deisotope	Yes	No
Chimeric spectra	No	No
Predict retention time	Yes	Yes
Number of peaks (min to max)	15 to 180	15 to 150
Minimum mached peaks	3	4
Maximum fragment charge	1	1
Wide window	Yes	No
Processing time	0:01:20	0:01:35
**# Identified proteins (PeptideShaker)**	1457	1113
**# Confident proteins (PeptideShaker)**	565	522
**# Unique peptides (PeptideShaker)**	3671	2790
**# Peptides (PeptideShaker)**	3936	3015
**# Identified spectra (PeptideShaker)**	**5362**	**4405**
**# Shared PSM**	**4269**	

Orange color highlights the mismatched parameters

(*) The parameter is set to "No" for search testing because of its effect on the searching time.

Confident proteins, peptides, and PSMs are calculated based on PeptideShaker analysis results

Detailed documentation for each search parameter is available at www.sage-docs.vercel.app/docs

Dataset 2 - PXD000561 (18.3MB) #8156 (Subset Mode: 3000)		
	**Subset Mode**	**PRIDE Mode**
Subset init time	0:00:33	00.00.00
Params picking time	0:02:40	00.00.00
**Parameters**		
Digestion	Enzyme	Enzyme
Enzyme	Trypsin	Trypsin
Specificity	Specific	Specific
Max missed cleavages	2	1
Fragment ion types	B,Y	B,Y
Precursor accuracy (PPM)	10	10
Fragment accuracy (Da)	0.02	0.05
Precursor charge	2 to 4	2 to 4
Isotops	0 to 1	0 to 1
Fixed modifications	Carbamidomethylation of C	Carbamidomethylation of C
Variable modifications	Deamidation of N<br>Methylation of K<br>Oxidation of M<br>Pyrolidone from Q	Oxidation of M<br>Acetylation of protein N-termini<br>Pyrolidone from Q
Peptide length (min-max)	7 to 30	8 to 30
Fragment Mz (min to max)	150 to 2000	200 to 2000
Peptide mass (min to max)	600.0 to 5000.0	600.0 to 5000.0
Min ion index	2	2
Maximum variable modifications	3	2
Generate decoy	Yes	No
Deisotope	Yes	No
Chimeric spectra	No	No
Predict retention time	Yes	Yes
Number of peaks (min to max)	10 to 100	15 to 150
Minimum mached peaks	3	4
Maximum fragment charge	1	1
Wide window	No	No
Processing time	0:00:40	0:00:06
**# Identified proteins (PeptideShaker)**	578	352
**# Confident proteins (PeptideShaker)**	221	188
**# Unique peptides (PeptideShaker)**	1334	924
**# Peptides (PeptideShaker)**	1526	1059
**# Identified spectra (PeptideShaker)**	**3353**	**2248**
**# Shared PSM**	**2167**	

Orange color highlights the mismatched parameters

(*) The parameter is set to "No" for search testing because of its effect on the searching time.

Confident proteins, peptides, and PSMs are calculated based on PeptideShaker analysis results

Detailed documentation for each search parameter is available at www.sage-docs.vercel.app/docs

Dataset 3 - PXD001468 (222MB) #55188 (Subset Mode: 3002)		
	**Subset Mode**	**PRIDE Mode**
Subset init time	0:01:34	00.00.00
Params picking time	0:06:03	00.00.00
**Parameters**		
Digestion	Enzyme	Enzyme
Enzyme	Trypsin	Trypsin
Specificity	Specific	Specific
Max missed cleavages	2	1
Fragment ion types	B,Y	B,Y
Precursor accuracy (PPM)	10	5
Fragment accuracy (Da)	0.02	0.02
Precursor charge	2 to 4	2 to 4
Isotops	0 to 1	0 to 1
Fixed modifications	Carbamidomethylation of C	Carbamidomethylation of C
Variable modifications	Deamidation of N<br>Deamidation of Q<br>Oxidation of M<br>Pyrolidone from Q	Oxidation of M
Peptide length (min-max)	5 to 30	8 to 30
Fragment Mz (min to max)	175 to 2000	200 to 2000
Peptide mass (min to max)	600.0 to 5000.0	600.0 to 5000.0
Min ion index	2	2
Maximum variable modifications	3	2
Generate decoy	Yes	No
Deisotope	Yes	No
Chimeric spectra	No	No
Predict retention time	Yes	Yes
Number of peaks (min to max)	15 to 200	15 to 150
Minimum mached peaks	4	4
Maximum fragment charge	1	1
Wide window	Yes	No
Processing time	0:00:59	0:00:10
**# Identified proteins (PeptideShaker)**	6803	4834
**# Confident proteins (PeptideShaker)**	3538	2732
**# Unique peptides (PeptideShaker)**	17860	11805
**# Peptides (PeptideShaker)**	18756	12331
**# Identified spectra (PeptideShaker)**	**35319**	**21879**
**# Shared PSM**	**21072**	

Orange color highlights the mismatched parameters

(*) The parameter is set to "No" for search testing because of its effect on the searching time.

Confident proteins, peptides, and PSMs are calculated based on PeptideShaker analysis results

Detailed documentation for each search parameter is available at www.sage-docs.vercel.app/docs

Dataset 4 - PXD047036 (251MB) #48032 (Subset Mode: 3002)		
	**Subset Mode**	**PRIDE Mode**
Subset init time	0:01:31	00.00.00
Params picking time	0:02:56	00.00.00
**Parameters**		
Digestion	Enzyme	Enzyme
Enzyme	Trypsin	Trypsin
Specificity	Specific	Specific
Max missed cleavages	2	2
Fragment ion types	B,Y	B,Y
Precursor accuracy (PPM)	10	20
Fragment accuracy (Da)	0.02	0.5
Precursor charge	2 to 4	2 to 4
Isotops	0 to 1	0 to 1
Fixed modifications	Carbamidomethylation of C	Carbamidomethylation of C
Variable modifications	Deamidation of N<br>Deamidation of Q<br>Oxidation of M<br>Pyrolidone from Q	Acetylation of protein N-termini<br>Oxidation of M
Peptide length (min-max)	7 to 30	8 to 30
Fragment Mz (min to max)	150 to 2000	200 to 2000
Peptide mass (min to max)	600.0 to 5000.0	600.0 to 5000.0
Min ion index	2	2
Maximum variable modifications	3	2
Generate decoy	Yes	No
Deisotope	Yes	No
Chimeric spectra	No	No
Predict retention time	Yes	Yes
Number of peaks (min to max)	15 to 180	15 to 150
Minimum mached peaks	3	4
Maximum fragment charge	1	1
Wide window	No	No
Processing time	0:01:02	0:00:22
**# Identified proteins (PeptideShaker)**	3910	1519
**# Confident proteins (PeptideShaker)**	1606	1008
**# Unique peptides (PeptideShaker)**	13543	6035
**# Peptides (PeptideShaker)**	14349	6426
**# Identified spectra (PeptideShaker)**	**25058**	**12097**
**# Shared PSM**	**10801**	

Orange color highlights the mismatched parameters

(*) The parameter is set to "No" for search testing because of its effect on the searching time.

Confident proteins, peptides, and PSMs are calculated based on PeptideShaker analysis results

Detailed documentation for each search parameter is available at www.sage-docs.vercel.app/docs

Dataset 5 - PXD009340 (91.8MB) #46084 (Subset Mode: 3002)		
	**Subset Mode**	**PRIDE Mode**
Subset init time	0:00:55	00.00.00
Params picking time	00:06:05	00.00.00
**Parameters**		
Digestion	Enzyme	Enzyme
Enzyme	Trypsin	Trypsin
Specificity	Specific	Specific
Max missed cleavages	2	2
Fragment ion types	B,Y	B,Y
Precursor accuracy (PPM)	10	10
Fragment accuracy (Da)	0.02	0.02
Precursor charge	2 to 4	2 to 4
Isotops	0 to 1	0 to 1
Fixed modifications	Carbamidomethylation of C	Carbamidomethylation of C
Variable modifications	Dimethylation of K<br>Oxidation of M<br>Pyrolidone from E<br>Pyrolidone from Q	Acetylation of protein N-termini<br>Oxidation of M
Peptide length (min-max)	8 to 30	8 to 30
Fragment Mz (min to max)	150 to 2000.	200 to 2000
Peptide mass (min to max)	600.0 to 5000.0	600.0 to 5000.0
Min ion index	2	2
Maximum variable modifications	2	2
Generate decoy	Yes	No
Deisotope	No	No
Chimeric spectra	No	No
Predict retention time	Yes	Yes
Number of peaks (min to max)	15 to 120	15 to 150
Minimum mached peaks	3	4
Maximum fragment charge	1	1
Wide window	No	No
Processing time	0:00:13	0:00:13
**# Identified proteins (PeptideShaker)**	3274	1982
**# Confident proteins (PeptideShaker)**	1610	1453
**# Unique peptides (PeptideShaker)**	12002	8734
**# Peptides (PeptideShaker)**	12804	9404
**# Identified spectra (PeptideShaker)**	**16138**	**11693**
**# Shared PSM**	**10973**	

Orange color highlights the mismatched parameters

(*) The parameter is set to "No" for search testing because of its effect on the searching time.

Confident proteins, peptides, and PSMs are calculated based on PeptideShaker analysis results

Detailed documentation for each search parameter is available at www.sage-docs.vercel.app/docs

Dataset 6 - PXD001250 (672MB) #168634 (Subset Mode: 3002)		
	**Subset Mode**	**PRIDE Mode**
Subset init time	0:03:16	00.00.00
Params picking time	00:04:18	00.00.00
**Parameters**		
Digestion	Enzyme	Enzyme
Enzyme	Trypsin	Trypsin
Specificity	Specific	Specific
Max missed cleavages	2	2
Fragment ion types	B,Y	B,Y
Precursor accuracy (PPM)	10	10
Fragment accuracy (Da)	0.02	0.02
Precursor charge	2 to 4	2 to 4
Isotops	0 to 1	0 to 1
Fixed modifications	Carbamidomethylation of C	Carbamidomethylation of C
Variable modifications	Deamidation of N<br>Deamidation of Q<br>Oxidation of M<br>Pyrolidone from Q	Acetylation of protein N-termini<br>Oxidation of M
Peptide length (min-max)	7 to 30	8 to 30
Fragment Mz (min to max)	175 to 1750	200 to 2000
Peptide mass (min to max)	600.0 to 5000.0	600.0 to 5000.0
Min ion index	2	2
Maximum variable modifications	3	2
Generate decoy	Yes	No
Deisotope	Yes	No
Chimeric spectra	No	No
Predict retention time	Yes	Yes
Number of peaks (min to max)	15 to 200	15 to 150
Minimum mached peaks	4	4
Maximum fragment charge	1	1
Wide window	No	No
Processing time	0:00:56	0:00:28
**# Identified proteins (PeptideShaker)**	9889	5826
**# Confident proteins (PeptideShaker)**	8006	4889
**# Unique peptides (PeptideShaker)**	74965	46236
**# Peptides (PeptideShaker)**	78985	48787
**# Identified spectra (PeptideShaker)**	**107958**	**74528**
**# Shared PSM**	**71154**	

Orange color highlights the mismatched parameters

(*) The parameter is set to "No" for search testing because of its effect on the searching time.

Confident proteins, peptides, and PSMs are calculated based on PeptideShaker analysis results

Detailed documentation for each search parameter is available at www.sage-docs.vercel.app/docs

Supplementary Table 6: Summary of the comparison of the tested datasets between Subset Mode and Full Data Mode.

X!Tandem (#PSMs)									
Datasets	Subset Mode	Full Data Mode	Intersection	Gained	Lost	PSMs Overall Gained	Subset Mode Time	Full Data Mode Time	Saved Time
Dataset 1	5024	5107	4945	79	162	-83	0:03:00	0:36:00	0:33:00
Dataset 2	3186	3227	3027	159	200	-41	0:02:00	0:28:00	0:26:00
Dataset 3	29569	29569	29569	0	0	0	0:04:30	6:00:00	5:55:30
Dataset 4	19816	20237	18656	1160	1581	-421	0:04:00	3:30:00	3:26:00
Dataset 5	10777	10776	10280	497	496	1	0:04:00	2:00:00	1:56:00
Dataset 6	91151	91771	85990	5161	5781	-620	0:06:00	26:27:00	26:21:00

Sage (#PSMs)									
Datasets	Subset Mode	Full Data Mode	Intersection	Gained	Lost	PSMs Overall Gained	Subset Mode Time	Full Data Mode Time	Saved Time
Dataset 1	5362	5204	5173	189	31	158	0:02:30	0:30:00	0:27:30
Dataset 2	3353	3408	3132	221	276	-55	0:04:00	0:27:00	0:23:00
Dataset 3	35319	31160	29917	5,402	1243	4159	0:08:00	0:46:00	0:38:00
Dataset 4	25058	25101	22442	2,616	2659	-43	0:04:00	0:44:00	0:40:00
Dataset 5	16138	15759	12204	3,934	3555	379	0:05:00	0:34:00	0:29:00
Dataset 6	107958	104783	100636	7,322	4147	3175	0:06:00	1:15:00	1:09:00

Supplementary Table 7: Summary of the comparison of the tested datasets between Subset Mode and PRIDE Mode.

X!Tandem (#PSMs)						
Datasets	Subset Mode	PRIDE Mode	Intersection	Gained	Lost	PSMs Overall Gaine
**Dataset 1**	5024	4946	4787	237	159	78
**Dataset 2**	3186	2769	2698	488	71	417
**Dataset 3**	29569	25769.0	23959	5610	1810	3800
**Dataset 4**	19816	12207	10871	8945	1336	7609
**Dataset 5**	10777	9428	8463	2314	965	1349
**Dataset 6**	91151	83719	78100	13051	5619	7432

Sage (#PSMs)						
Datasets	Subset Mode	PRIDE Mode	Intersection	Gained	Lost	PSMs Overall Gaine
**Dataset 1**	5362	4405	4269	1093	136	957
**Dataset 2**	3353	2248	2167	1186	81	1105
**Dataset 3**	35319	21879	21072	14247	807	13440
**Dataset 4**	25058	12097	10801	14257	1296	12961
**Dataset 5**	16138	11693	10973	5165	720	4445
**Dataset 6**	107958	74528	71154	36804	3374	33430
